# Targeted and Non-Targeted Screening of Organic Pollutants in Atmospheric Aerosols of Arctic Urban Agglomeration Using TD-GC-Orbitrap MS

**DOI:** 10.3390/molecules31101636

**Published:** 2026-05-13

**Authors:** Irina S. Shavrina, Kirill O. Sukhanov, Nikolay V. Ul’yanovskii, Dmitry S. Kosyakov, Albert T. Lebedev

**Affiliations:** 1 Laboratory of Environmental Analytical Chemistry, Core Facility Center “Arktika”, Northern (Arctic) Federal University, 163002 Arkhangelsk, Russia; i.shavrina@narfu.ru (I.S.S.); suhanov.k.o@edu.narfu.ru (K.O.S.); n.ulyanovsky@narfu.ru (N.V.U.); 2 Department of Materials Science, MSU-BIT University, Shenzhen 517182, China; a.lebedev@org.chem.msu.ru

**Keywords:** air pollution, organic pollutants, particulate matter, thermal desorption, gas-chromatography, mass spectrometry

## Abstract

This study focuses on the search and determination of organic pollutants in aerosol particles (PM_2.5_) collected in Arkhangelsk, the largest urban agglomeration in the Arctic, during winter and summer periods. Thermal desorption gas chromatography coupled with high-resolution mass spectrometry was applied for non-targeted screening of atmospheric aerosols, enabling the detection of compounds at low concentrations ranging from 10 pg/m^3^ to several ng/m^3^. Representatives of various chemical classes were detected in samples from both seasons, including CHO compounds (with phthalates as the predominant subgroup), nitrogen-containing compounds (e.g., pyridines, quinolines, nicotine), phenols and monoaromatics, as well as polycyclic aromatic hydrocarbons and their oxygenated derivatives. In winter, PAHs and oxy-PAHs significantly predominated, likely due to increased combustion of fossil fuels and biomass for heating purposes. A total of approximately 300 compounds were identified via non-targeted screening, of which 32 were confirmed and quantified using authentic reference standards across six chemical classes. Seasonal variations in both the composition and concentration levels highlight the impact of local emission sources and atmospheric conditions on the organic aerosol profile in this arctic urban environment.

## 1. Introduction

Arctic pollution has attracted the attention of researchers for several decades. Despite low population density and the remoteness of the region from major industrial centers, contaminants are consistently found in the air, snow, and soil [1].

An important separate criterion for assessing atmospheric air quality is the level of aerosol particles. The concentration of aerosols in the Arctic atmosphere varies seasonally, with a consistent pattern of increased particle levels in winter and spring [2]. Arctic aerosol particles may originate from local natural and anthropogenic sources (transport, mineral extraction, etc.). A significant factor contributing to the increase in aerosol levels is the long-range transport effect, whereby anthropogenic emissions from urban environments in European and Asian countries, as well as biomass burning in Siberia, become the primary sources of pollutants [3,4]. Consequently, it becomes important to investigate the atmospheric pollution of Arctic cities, which have the potential to act as sources of pollutants to the Arctic region.

The increase in atmospheric aerosol particles has a substantial impact on the Arctic ecosystem, affecting surface air temperature and the deposition of black carbon on snow and ice surfaces [5]. Furthermore, the danger of atmospheric aerosols lies in their ability to adsorb more toxic substances onto their surfaces, potentially causing serious impacts on sensitive Arctic ecosystems and, when inhaled, affecting human health. The organic fraction of airborne particulate matter comprises thousands of compounds, ranging from simple hydrocarbons to highly oxidized species [6]. Studying its composition is an extremely complex task, in which a crucial step is the selection of the analyte preconcentration method. The most common technique is Soxhlet extraction [7]. Despite its advantages (simplicity of operation, low cost), this method requires lengthy extraction times and large volumes of solvent. Alternative methods include pressurized liquid extraction [8], microwave-assisted extraction [9], ultrasonic extraction [7], and solid-phase microextraction [10]. Despite the prevalence of various extraction methods, this approach has several drawbacks, such as labor-intensive processes, the use of toxic solvents, potential errors leading to analyte loss, and the requirement for large sample volumes.

Therefore, there is a clear need for a solvent-free, sensitive, and comprehensive analytical approach for characterizing the organic composition of Arctic aerosols. Thermal desorption gas chromatography—high-resolution mass spectrometry (TD-GC-HRMS) offers a promising alternative, combining minimal sample preparation with the resolving power required for non-target screening [11]. An additional advantage of this method is the small sample amount required for analysis, which is particularly crucial for studies in remote and hard-to-reach Arctic regions.

Thermal desorption GC-MS has already proven itself as a promising approach for the targeted analysis of organic pollutants in atmospheric particulate matter [12,13,14,15]. Over the past decade, several studies have focused on optimizing TD methods for the targeted analysis of a range of common contaminants [11,16,17,18]. However, this method has not yet been applied for the comprehensive characterization of the organic fraction of atmospheric aerosols. The combination of thermal desorption with high-resolution mass spectrometry provides a powerful analytical tool, enabling more confident identification of unknown compounds through accurate mass measurements and the determination of corresponding molecular formulas, which is essential for non-target screening in complex environmental matrices.

Therefore, the aim of this study was to expand the application area of direct thermal desorption GC-HRMS and characterize the chemical composition of organic pollutants in PM_2.5_ samples collected from Arkhangelsk, a major urban agglomeration in the Russian Arctic. The study focused on the simultaneous non-targeted screening and targeted determination of a wide range of organic contaminants, including CHO compounds (with phthalates), nitrogen-containing heterocycles, phenols, PAHs, oxy-PAHs, and halogenated compounds. In addition, seasonal variations in the chemical composition and the predominant pollution sources were assessed.

## 2. Results and Discussion

### 2.1. Method Description and General Characteristics of the Samples

The proposed analytical methodology is based on direct thermal desorption with gas chromatography coupled to high-resolution mass spectrometry for targeted and untargeted screening of organic pollutants in PM_2.5_. Preliminary experiments comparing glass and quartz fiber filters demonstrated several advantages of the former for the chosen analytical method. Both filter types are stable at typical thermal desorption temperatures (up to 320 °C). However, glass fiber filters showed lower background contamination levels. Furthermore, they were more durable and thinner, facilitating their placement in the thermal desorption tube—an important factor given that the entire filter was analyzed. Based on these results, glass fiber filters were selected for all subsequent sampling.

A key advantage of the TD-GC HRMS method is its high sensitivity, achieved with a relatively small sample volume. The optimal sampling rate and duration for urban aerosol sampling using a 47 mm GF/A filter are 15 L/min and approximately 2 h. Detection limits for most target compounds range from 10 pg/m^3^ to several ng/m^3^, achieved with a sample volume of only 1.8 m^3^ of air. This sample volume is significantly smaller than typically required in traditional air monitoring studies (often hundreds or thousands of m^3^), yet still provides sufficient sensitivity for assessing urban air quality. This makes the method particularly suitable for future studies in remote, inaccessible areas of the Arctic. However, for some analytes, such as 3-nitroaniline, the limit of quantification was higher (20 ng/m^3^) due to lower ionization efficiency.

The thermal desorption parameters used in this study (300 °C, 15 min) were optimized for PAHs and oxy-PAHs. While no significant degradation or carryover was observed for these compound classes, the stability of other groups (phthalates, phenols, and CHO compounds) under the same conditions was not systematically tested. Potential thermal artifacts—such as partial degradation of labile compounds or formation of oxidation products—cannot be completely excluded.

The duration of the second desorption (desorption from a cold-focus trap) was set to 5 min, during which the analytes were transferred to the GC column, which operated in a non-separation mode with a pressure pulse. To assess the completeness of thermal desorption, an additional desorption step was performed immediately after the first one for selected filters. For both the target and non-target compounds, no detectable signals were observed in the second TD run, confirming that the single desorption step quantitatively transferred the analytes from the filter.

**Figure 1:**
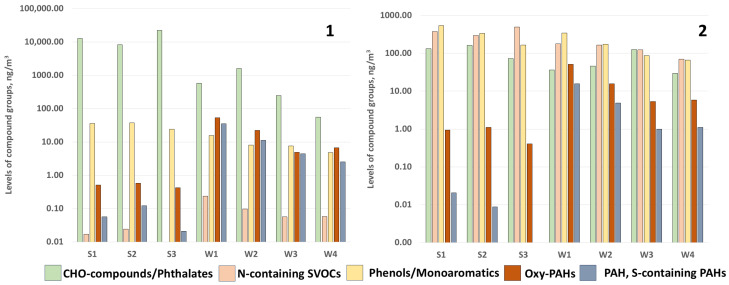
Levels of the main pollutant group detected in the samples: 1—compounds confirmed with authentic standards (targeted analysis); 2—compounds tentatively identified via non-targeted screening (semi-quantitative estimates).

Given the chemical complexity of the samples, the initial untargeted screening identified a large number of compounds (an average of 1500). To focus our discussion on the most reliable results, we prioritized compounds with high identification rates (>80%). The list of detected compounds was manually verified to ensure mass accuracy (<5 ppm) and confirm assigned molecular formulas. Furthermore, only compounds with peak areas at least an order of magnitude greater than those of the corresponding control sample were selected for further analysis. A total of approximately 300 compounds were detected across all samples (Table S2). Among these, 32 were unequivocally confirmed using analytical standards and quantified. The identified compounds fell into five main chemical classes: CHO-compounds, including phthalates, *N*-containing compound, phenols and monoaromatic compound, polycyclic aromatic hydrocarbons (PAHs) and their oxygenated derivatives (oxy-PAHs) (Figure 1). It should be noted that several environmentally significant classes of compounds, including polychlorinated biphenyls, per- and polyfluoroalkyl substances, and nitrated polycyclic aromatic hydrocarbons, were not detected during non-targeted screening under the applied conditions. This lack of detection may be due to either methodological limitations (the EI is not optimal for some classes of compounds) or concentrations below the detection limits of the method.

CHO compounds were the most numerous group in all samples, mainly due to the high content of phthalates. Their total concentration reached 22 μg/m^3^ in one of the warm period samples. A seasonal trend was also observed for polycyclic aromatic hydrocarbons and their oxygenated derivatives, which predominated during the winter period. The total content of nitrogen compounds in both winter and summer is in the region of several hundred ng/m^3^. The number of samples is limited (four and three for winter and summer, respectively). Therefore, the observed seasonal patterns should be interpreted as preliminary, and further studies with more frequent systematic sampling are required to confirm these trends.

The concentrations of key pollutant classes in the present study are consistent with previously reported data from Arctic and subarctic regions. Phthalate levels (summer: 0.2–15 µg/m^3^, winter: 0.005–1.0 µg/m^3^) are comparable to those observed in ambient air from the Russian Arctic [19], where phthalates were also reported as dominant pollutants. Phenol concentrations (5 ng/m^3^) are in agreement with levels reported in Arctic air and snow, where phenols were detected as major contaminants [19,20]. PAH concentrations (winter: up to 50 ng/m^3^, summer: <1 ng/m^3^) and the predominance of fluoranthene and pyrene correspond well with earlier studies in Arkhangelsk snow and aerosols [21,22]. The winter maximum of PAHs is consistent with pyrogenic sources (coal and biomass combustion) dominating the cold season, as previously observed in Arctic atmospheric studies [23,24]. Overall, the data obtained in this study show good general agreement with existing literature, supporting the validity of our findings despite the limited number of samples.

### 2.2. Targeted Analysis and Quantification of Pollutants

The targeted analysis was conducted using 72 reference standards. Thirty-two of these were detected and quantified in the samples, while the remaining 40 were below detection limits (Table 1). The detected compounds represent five groups: nitrogen-containing heterocycles, phenols (including halogenated phenols), polycyclic aromatic hydrocarbons, their oxygen-containing derivatives, and phthalates.

As expected, given their widespread industrial use and presence in all environmental samples, even in cloud water [25] and Arctic snow [26] and air [19], phthalates were the dominant class in all samples. Concentrations ranged from 8.1 to 22 µg/m^3^ in summer and from 0.050 to 1.5 µg/m^3^ in winter, with summer levels exceeding winter levels by approximately an order of magnitude. This seasonal pattern is consistent with previous studies showing that an increase in ambient temperature can increase their emission rates from plastic products [27,28].

In terms of total concentration of target analytes, phenols rank second after phthalates. This group is represented by phenol (5–6 ng/m^3^) and its methyl derivatives, о-cresol (0.51–10 ng/m^3^), m, p-cresols (0.55–20 ng/m^3^). Benzyl alcohol, which was classified in this group, was also detected in concentrations ranging from 0.93 ng/m^3^ to 1.4 ng/m^3^. 2,4-Dichlorophenol was the only representative of the halogenated semi-volatile pollutants. These compounds likely originate from biomass combustion, industrial activity, and vehicle exhaust [29,30]. It was previously established that phenols are one of the main classes of pollutants in Arkhangelsk snow, with concentrations reaching up to 300 ng/L [31].

In contrast to phthalates, target PAHs and oxy-PAHs showed an inverse seasonal relationship, with a maximum concentration of 60 ng/m^3^ in winter, while in summer, levels did not exceed 1 ng/m^3^. Among the PAHs, the predominant pollutants were fluoranthene and pyrene (8 ng/m^3^ each). Earlier studies of snow from Arkhangelsk [21,31], Moscow [32] and samples from Arctic regions [20,33] confirmed that fluoranthene and pyrene are among the main representatives of PAHs in the environment. Low-molecular-weight PAHs, acenaphthylene and dibenzofuran, were detected in quantities of several hundred pg/m^3^. Concentrations of carcinogenic benzo[a]pyrene ranged from 0.21 to 1.6 ng/m^3^, exceeding the maximum allowable concentration (1 ng/m^3^) [34] in one winter sample. Our previous study showed a similar distribution of PAHs in airborne aerosols, where the major sources were identified as pyrogenic processes (incomplete fuel combustion and biomass burning) based on diagnostic ratios (Table S2) [22]. Consistent with this, PAHs and oxy-PAHs were significantly more prevalent in winter, likely due to increased combustion of fossil fuels and biomass for heating.

The vast majority of target nitrogen-containing compounds were not detected in the air samples analyzed. The exception is carbazole, a toxic substance included on the list of priority pollutants. It was detected above the LOQ in five aerosol samples, with average concentrations of approximately 50 pg/m^3^. Carbazoles and their halogenated derivatives are considered potentially hazardous, persistent, and bioaccumulative pollutants. They enter the environment may be associated with waste incineration, biomass burning, rubber, oil, and coal combustion, as well as various industrial processes [35,36,37].

### 2.3. Non-Targeted Screening and Semi-Quantification of Pollutants

To comprehensively characterize the organic aerosol composition, non-targeted (unknown unknown) screening was conducted. Detected compounds were subsequently categorized into chemical classes. Compound identification was performed using TraceFinder 5.0. The software calculates several metrics: SI (search index, 0–999), RSI (reverse search index, 0–999), HRF score (% of total ion current explained by the chemical formula), RHRF score (reverse high-resolution filtering), and a combined Score (0–100). Only compounds with a combined Score ≥ 80 were retained for further analysis. Compounds with lower scores were excluded from the dataset. Table 2 presents the most abundant or frequently detected compounds from each chemical group identified through non-target screening. A complete list of all detected compounds is provided in Table S3.

*Nitrogen-containing pollutants*. The largest number of detected compounds was nitrogen-containing pollutants. This group included a wide range of substances, such as methyl-substituted pyridines, quinolines and their derivatives, nicotine, and others. The approximate levels of these compounds ranged from 69 to 489 ng/m^3^. Among them, several compounds showed semi-quantitative levels above 20 ng/m^3^, including caprolactam, *N*-butylbenzenesulfonamide, diethyltoluamide (DEET), nicotine, and nicotyrene.

The detection of *N*-butylbenzenesulfonamide and caprolactam in our aerosol samples is consistent with previous PM_2.5_ studies, where their presence was attributed to their widespread use in the production of nylon products and other plastics [38,39]. In our study, these compounds were detected at levels from 2.3 to 204 ng/m^3^, with higher levels similar to phthalates observed in summer, which once again confirms their origin from polymer-based products.

The presence of DEET in the samples is likely associated with its use as an insect repellent. Notably, this compound was also detected in winter (0.93–3.8 ng/m^3^), although its concentrations were an order of magnitude higher in summer (12–240 ng/m^3^). DEET has been repeatedly detected in air, wastewater, and snow samples, and wastewater treatment plants have been proposed as a significant source of its year-round occurrence in the atmosphere [40,41,42]. In Arctic areas, DEET has been detected in atmospheric aerosols [43], seawater samples [44], and snow [45], where it is possible that atmospheric precipitation is a likely mechanism for the transport of DEET over local, regional, or long distances, but this process has not been fully studied.

Nicotine, with estimated concentrations in the samples ranging from 0.5 to 45 ng/m^3^, is the most common organic compound released during smoking [46]. Its derivatives, including methyl nicotinate, 3-(3,4-dihydro-2H-pyrrol-5-yl)pyridine, nicotyrine, and 4,4′-bipyridine, are also likely pollutants that may originate from cigarette smoke. The average concentration of these compounds was approximately 0.5 ng/m^3^.

**Figure 2:**
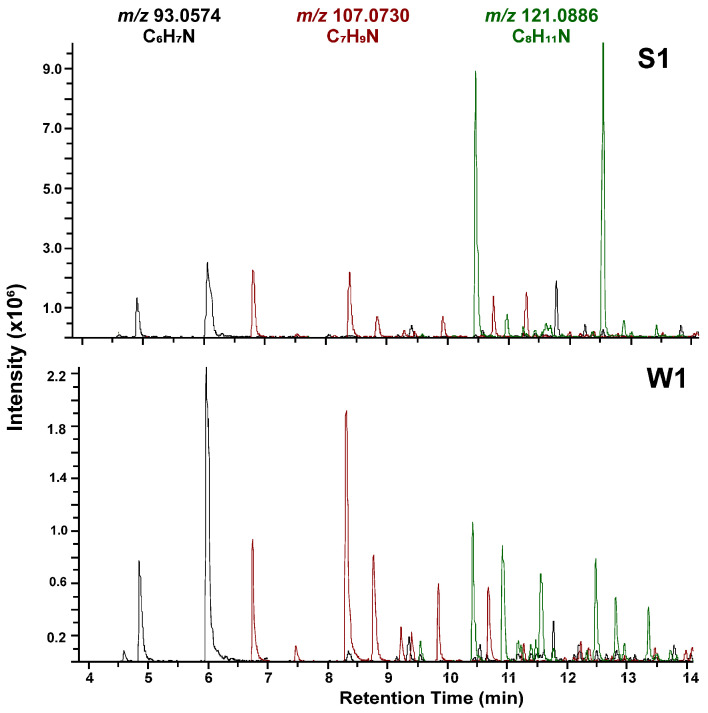
Reconstructed XIC chromatogram of the methylpyridines in aerosol samples S1 and W1 with a mass accuracy window of ±5 ppm.

Methyl-substituted pyridines with concentrations from 1 to 5 ng/m^3^ can be classified as a separate subgroup. Sources of this group of pollutants may include the aforementioned cigarette smoke [47], as well as biomass and peat combustion processes [48]. Among these, C_1_, C_2_, and C_3_-substituted pyridines were identified (Figure 2). In the extracted ion chromatograms based on exact masses, approximately 10–13 intense peaks were observed, along with less intense peaks that could not be identified with sufficient confidence. However, these may represent potentially hazardous pollutants, and their structures will be elucidated in future studies. For several years, non-targeted analysis of environmental samples has identified pyridine and its derivatives in various samples: snow collected in Arkhangelsk [31], Arctic snow from the Novaya Zemlya archipelago [20], rainwater in Moscow [49] and cloud water in central France [25]. However, it is surprising that there are not many studies mentioning the detection of pyridines in atmospheric aerosol samples. This may be due to the fact that most studies mainly focus on targeted analysis.

Quinolines, benzoquinolines, and their derivatives represent another subgroup of nitrogen-containing pollutants. These compounds are commonly referred to as azaarenes—polycyclic aromatic compounds in which a carbon atom in the aromatic ring is replaced by a nitrogen atom. These compounds have been detected in atmospheric particulate matter in various regions, including Southern European cities [50], France [51], China [52], and Russia [22,53]. Their sources are likely similar to those of PAHs, potentially including vehicle exhaust, coal combustion, and biomass combustion. Azaarenes concentrations reported in the literature vary by location and season, with typical levels in the 10 ng/m^3^ range, while in our study, they ranged from 3 ng/m^3^ in the sample S3 to 92 ng/m^3^ in the sample W1.

**Figure 3:**
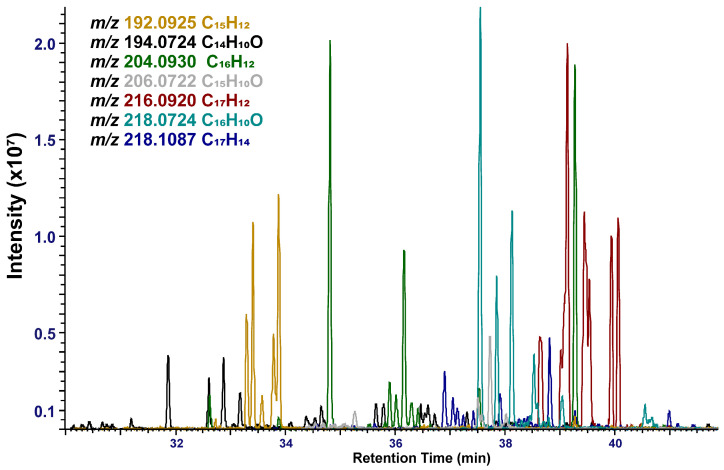
Reconstructed XIC chromatogram of the PAHs and oxy-PAHs in aerosol sample W1 with a mass accuracy window of ±5 ppm.

*PAHs and oxy-PAHs*. Identifying individual PAHs and their derivatives presented a challenging task due to their structural similarity and frequent co-elution on the chromatography column. Many isomers exhibited nearly identical mass spectra and partially overlapping retention times, resulting in complex peak clusters in the extracted ion chromatograms (Figure 3). To overcome these limitations, comprehensive two-dimensional gas chromatography would be useful. This may be the focus of our future research, as it provides improved separation of structurally similar isomers, allowing for more reliable identification and quantification of individual PAHs [54].

**Figure 4:**
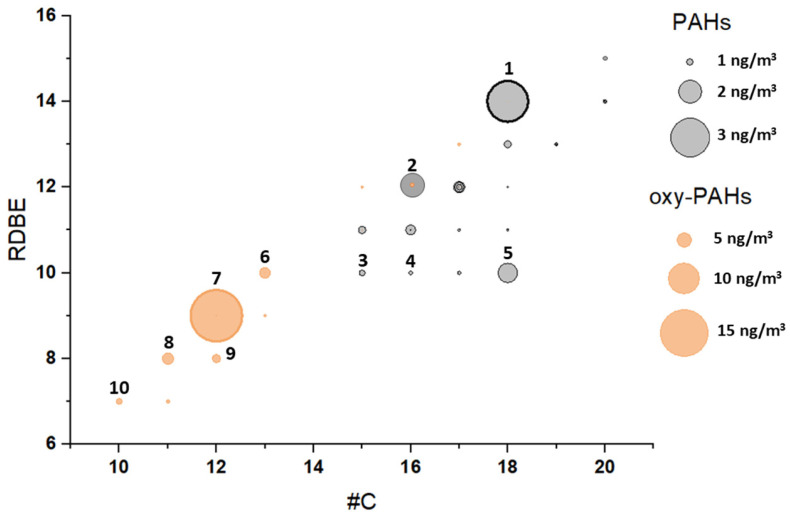
Chemical composition of the PAHs and oxy-PAHs in the W1 sample visualized in double-bond equivalent—number of carbon atoms plot, and bubble size corresponds to semi-quantitative concentrations (ng/m^3^) estimated using the calibration curve of the nearest structural analog. The numbered bubbles are keyed to the molecular structures in Figure 5.

Studies have already shown that, in addition to the 16 priority PAHs included in the EPA list, a large number of other polyaromatic compounds can be identified in atmospheric samples [55,56]. Many of these compounds, including alkylated PAHs, oxygenated PAHs, and heterocyclic derivatives, may have higher mutagenic and carcinogenic potential than their parent compounds. In our study, non-target screening identified a wide range of PAHs and oxy-PAHs. These compounds ranged from C15 to C20, and they contained from 2 (RDBE 7) to 5 (RDBE 15) aromatic rings (Figure 4). Seasonal fluctuations were observed for both targeted PAHs and compounds identified through untargeted screening. In winter, the total number of detected PAHs and oxy-PAHs reached approximately 80, compared to only 10–15 in summer.

**Figure 5:**
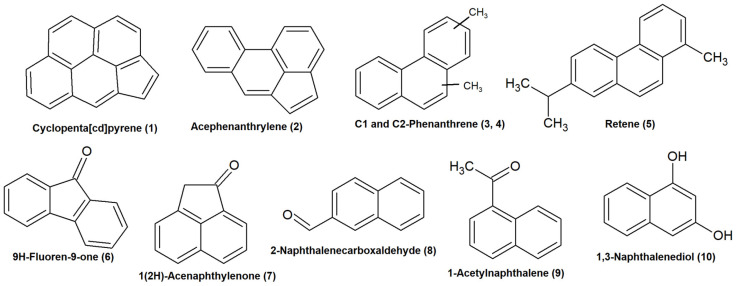
Chemical structures of main PAHs and oxy-PAHs, identified in aerosol samples.

Figure 5 shows the main representatives of this group of pollutants. Only a few compounds showed estimated concentrations above 1 ng/m^3^—acephenanthrylene, retene, and cyclopenta[cd]pyrene. Of the alkyl derivatives, the most common are C_1_, C_2_-phenanthrenes, followed by C_1_-pyrenes. Among the oxy-PAHs, naphthalene derivatives—1,3-naphthalenediol, 1-(1-naphthalenyl)-ethanone—deserve special mention. They were identified in all aerosol samples. The level of 1(2H)-acenaphthylenone reached 17 ng/m^3^ in one of the samples. In addition, two PAHs with a sulfur heteroatom, presumably thioxanthene and benzo[b]naphtho [1,2-d]thiophene, were identified in amounts of about several pg/m^3^.

*CHO compounds*. Most of the identified representatives of this group can be classified as oxygen-containing organic compounds, i.e., oxidation products of hydrocarbons. The overall level ranged from 30 to 162 ng/m^3^, with no clear seasonal trends observed.

Furan derivatives were detected in all samples. This group of compounds is typically associated with the degradation of cellulose and other carbohydrates during biomass combustion or pyrolysis, and its representatives have even been detected in Arctic snow samples [20]. Several fatty acid esters (propanoic, butanoic, and hexadecanoic acids) were also identified, which may originate from biogenic lipid hydrolysis or oxidation of aliphatic hydrocarbons.

*Phenols and monoaromatics*. Aromatic compounds such as benzaldehyde derivatives, methoxy derivatives of benzene and phenol, and alkylbenzenes were identified in each analyzed sample, with estimated concentration ranging from 66 to 550 ng/m^3^. Several of these compounds—including 4-hydroxybenzaldehyde, vanillin, apocynin, 1-(3-hydroxy-4-methoxyphenyl)ethanone, 1-(4-hydroxy-3-methoxyphenyl)propan-2-one, and 4-hydroxy-3,5-dimethoxybenzaldehyde—can be classified as products of wood combustion formed during lignin degradation [57]. As shown in previous studies [58,59,60,61,62,63,64,65], phenols can undergo atmospheric oxidation, nitration, and photochemical conversion reactions, especially in multiphase systems such as aerosols. Although such compounds are most often considered markers of wood combustion or industrial emissions, their concentrations can be altered by secondary atmospheric processes.

## 3. Materials and Methods

### 3.1. Sample Collection

**Figure 6:**
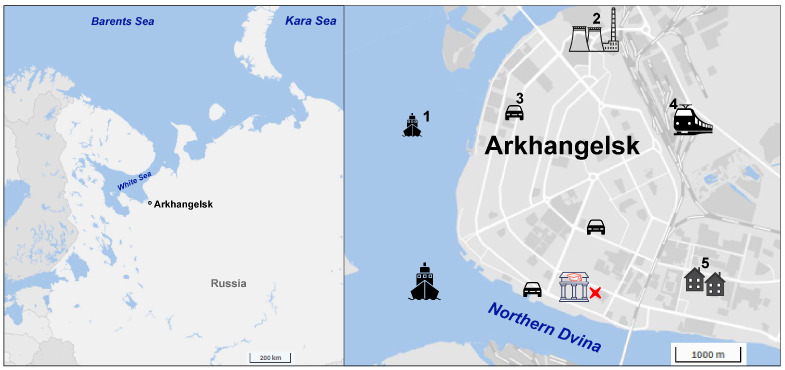
Sampling site location in Arkhangelsk (red marker), northwestern Russia and main local emission sources (1—shipping, 2—power plant, 3—vehicle exhaust emission, 4—railway line, 5—residential heating).

The study was conducted in Arkhangelsk, a large Arctic city in northwestern Russia (population ~300,000), located on the banks of the Northern Dvina River (Figure 6). The city is characterized by a mix of local emission sources, including vehicle traffic, a pulp and paper mill (approximately 25 km from the sampling site), a coal- and gas-fired combined heat and power plant, and residential wood combustion during winter [21]. The sampling site is located in a mixed residential and educational area near a highway with moderate traffic density.

PM_2.5_ samples were collected on GF/A glass fiber filters (Whatman, Maidstone, UK) using an air sampler (PU-4E, Chimko, Moscow, Russia) on the rooftop (approximately 5 m above ground level) of a building at the Northern (Arctic) Federal University in Arkhangelsk, Russia, during the winter of (December 2024: W1, W2; January 2025: W3, W4) and summer (August 2025: S1, S2, S3) periods. The samples were collected for 2 h at a flow rate of 15 L/min, after which the filters were frozen at −20 °C. The filters were pre-cleaned by heating at 450 °C for 24 h in a muffle furnace to remove organic impurities. Meteorological conditions (temperature, humidity, wind speed, and wind direction) for each sampling date are summarized in Table S4. Field blanks (*n* = 2) were collected during winter and summer campaigns and were stored together with the sample filters under identical conditions (−20 °C) prior to analysis. Each field blank was handled identically to the samples but was exposed to ambient air for 30 s without active sampling. One laboratory blank (pre-cleaned filter without field exposure) was also analyzed.

### 3.2. Thermal Desorption Gas Chromatography—High-Resolution Mass Spectrometry

All experiments were performed using a Unity-xr thermal desorber (Markes, Bridgend, UK) coupled with an Exactive GC gas chromatography—mass spectrometry system (Thermo Scientific, Waltham, MA, USA), which consisted of a Trace 1310 gas chromatograph and an Orbitrap high-resolution mass spectrometer. A two-step thermal desorption technique was used to extract organic pollutants from the aerosol filter surface. The filter was folded and placed into an empty pre-conditioned stainless steel thermal desorption tube (3.5 × ¼ inch, Markes, Bridgend, UK) and manually loaded into the TD system. Before heating, the sorption tube with the sample was purged with a carrier gas (helium, 99.9999%, NIIKM, Moscow, Russia) at a flow rate of 50 mL/min for 1 min to remove excess moisture interfering with GC separation. At the first stage, the tube was rapidly heated to 300 °C and held for 15 min at a helium flow rate of 50 mL/min to desorb analytes and focus them on a cold (10 °C) “PAH” sorption trap. A splitless mode was used. At the second stage, the sample was introduced into the GC column by rapid heating of the trap to 320 °C (held for 5 min), also in splitless mode. Sample injection was performed with a pressure pulse of 200 kPa, corresponding to a flow rate of 3 mL/min.

Chromatographic separation was achieved using a TG-5SilMS fused silica capillary column (Thermo Scientific, Waltham, MA, USA) with dimensions of 30 m × 0.25 mm and a film thickness of 0.25 μm, coated with a stationary phase consisting of (5% phenyl)-methylpolysiloxane. The oven temperature program was as follows: initial temperature of 30 °C was held for 1 min, then increased to 300 °C at a rate of 5 °C/min, and held for 6 min. The total run time was 60 min. The ion source and GC-MS transfer line were maintained at 250 °C and 300 °C, respectively.

Mass spectrometric detection was carried out in full scan mode over an *m*/*z* range of 40–500, with a resolving power set to 30,000 (FWHM at *m*/*z* 200) and an acquisition frequency of 12 Hz. Electron ionization (EI) at 70 eV was employed. Mass calibration was performed daily using perfluorotributylamine (PFTBA) as a reference standard, ensuring mass accuracy below 3 ppm (typically less than 1 ppm). Automatic gain control (AGC) was applied with a target filling value of 1 × 10^5^ ions for the C-Trap. The Xcalibur 4.1 software (Thermo Scientific, Waltham, MA, USA) was used for system control, data acquisition, and preprocessing. Only compounds exhibiting a signal-to-blank ratio greater than 10 were considered as analytes for subsequent quantification and non-target screening.

### 3.3. Targeted Analysis

Seventy-two semi-volatile organic compounds were selected as target analytes (Table S1). Quantification was carried out using external calibration with a commercially available EPA Method 8270 MegaMix standard mixture (Restek, Bellefonte, PA, USA) for 64 compounds, and additional analytical standards for oxy-PAHs (Macklin, Shanghai, China). Compound identification was confirmed by retention time, accurate mass, and mass spectral matching. Calibration solutions in the concentration range of 0.01–10 ppm were prepared by sequential dilution of the standard mixture (1000 mg/L) with methanol (HPLC gradient grade, Khimmed, Moscow, Russia). Aliquots of the calibration solutions were spiked directly onto pre-cleaned GF/A filters using a microsyringe, and the filters were then placed into sorption tubes. Because the filter itself constitutes the primary matrix and the mass of collected PM_2.5_ is negligible compared to the filter mass, the filter-based calibration partially accounts for matrix effects arising from the filter material. However, we recognize that real aerosol constituents may influence desorption behavior and artefact formation. The TD tubes were subsequently desorbed and analyzed under the same conditions as the samples. Carryover was assessed by analyzing a blank filter immediately after a high-concentration standard (1 µg/mL). No significant peaks were detected in the blank; the residual signal for any target compound was below 0.1% of the standard peak area (Figure S2). The stability and repeatability of the analytical system were tested via thermal desorption analysis of three repetitions with a component amount of 5 ng per filter. The relative standard deviations (RSDs) for the peak areas of the target compounds were 10%.

The most intense peaks in the mass spectra of the analytes (predominantly corresponding to molecular ions [M]^+^•) were used for quantification. To ensure high selectivity, sensitivity, and low background noise, a narrow *m*/*z* window (5 ppm) was applied for extracting ion current (XIC) chromatograms. TraceFinder 5.0 software (Thermo Scientific, Waltham, MA, USA) was used to construct calibration plots and calculate the masses of the analytes. Instrumental limits of detection (LODs) and quantification (LOQs) were estimated using signal-to-noise ratio criteria of 3 and 10, respectively. The obtained values (Supplementary Table S1) ranged from 0.003 to 11 ng on-column for most analytes.

### 3.4. Non-Targeted Screening

The non-target analysis procedure involved peak finding, deconvolution of the overlapped chromatographic peaks, thus obtaining clean EI mass spectra, and generating a list of mass spectral library hits with corresponding match scores using a TraceFinder 5.0 software (Thermo Scientific, Waltham, MA, USA) with an additional deconvolution plugin. The ion overlap window was set to 98%, meaning that extracted ion chromatograms were considered to belong to the same compound only if their peak shapes overlapped by at least 98% in retention time. Similarity index (SI) and peak intensity thresholds were 500 and 1 × 10^5^, respectively. The mass tolerance for all compounds was 5 ppm and the signal-to-noise ratio ≥ 3. The NIST-2020 and Wiley-11 spectral libraries were used to identify unknown compounds found in the samples. In the case of compounds with low SI, the exact mass and EI fragmentation rules were used to elucidate the chemical formula and molecular structure. The concentrations of non-target compounds were estimated based on the calibration curves of the nearest structurally related compounds from the targeted analysis with similar retention times.

## 4. Conclusions

A combination of non-target screening and target analysis of PM_2.5_ atmospheric samples from Arkhangelsk revealed the presence of approximately 300 organic pollutants. Comprehensive target screening was conducted on PM_2.5_ samples from an Arctic city, covering such diverse groups of compounds as nitrogen-containing heterocycles, halogenated compounds, polycyclic aromatic hydrocarbons, their oxygenated derivatives, and phthalates. Phthalates, as well as methyl- and dimethyl-substituted phenols, were among the most prevalent compound classes. Significant seasonal variability was observed for all PAHs, with the highest concentrations occurring in winter, when combustion sources are most active. Fluoranthene and pyrene concentrations reached 8 ng/m^3^ each, while the concentration of the carcinogenic benzo[a]pyrene was 1.6 ng/m^3^ in one winter sample, exceeding the maximum permissible concentration. The most abundant oxy-PAHs in the aerosol samples were 1(2H)-acenaphthylenone, 9H-fluoren-9-one, hydroxybiphenyl isomers, anthraquinone, and anthrone. Mono-, di-, and trimethylpyridines represent a potentially hazardous class of compounds identified in PM samples at concentrations of several ng/m^3^.

This study demonstrates that the combination of direct thermal desorption with high-resolution mass spectrometry can effectively support both target screening and the discovery of novel pollutants in urban environments. Future studies should focus on systematic validation of the TD-GC-HRMS method for less stable compound classes, application of modeling for quantitative source apportionment. Extending the method to remote Arctic sites would further demonstrate its value for polar atmospheric research.

## Figures and Tables

**Table 1:** Concentration of target pollutants in the samples.

No.	tr, min	Compounds	Concentration, ng/m^3^						
			S1	S2	S3	W1	W2	W3	W4
*N*-compounds									
1	32.33	Carbazole	<0.021	0.024	-	0.023	0.097	0.056	0.057
Phenols, benzyl alcohol									
2	10.28	Phenol	6.6	5.8	4.5	-	-	-	-
3	11.89	Benzyl alcohol	1.4	1.2	0.93	1.1	-	-	-
4	12.62	2-Methylphenol	8.7	10	4.7	2.5	0.51	5.2	1.3
5	13.29	3+4-Methylphenol	17	20	13	10	6.3	0.55	0.170
6									
7	14.57	Isophorone	0.76	0.29	0.17	0.22	0.12	0.55	0.16
8	15.45	2,4-Dimethylphenol	1.2	0.87	0.63	1.4	1.2	1.2	3.1
9	15.96	2,4-Dichlorophenol	-	0.55	0.53	-	-	-	-
Polycyclic aromatic hydrocarbons									
10	23.55	Acenaphthylene	-	-	-	0.29	0.28	0.82	0.13
11	25.2	Dibenzofuran	-	-	-	0.13	0.080	0.42	0.039
12	30.95	Phenanthrene	-	-	-	2.4	0.30	0.19	0.22
13	31.27	Anthracene	-	-	-	0.33	0.062	0.031	0.044
14	36.45	Fluoranthene	-	-	-	8.0	1.3	0.5	0.43
15	37.39	Pyrene	-	-	-	8.2	1.4	0.6	0.43
16	43.06	Benz[a]anthracene	<0.10	<0.10	<0.10	4	1.8	0.27	0.36
17	43.22	Chrysene	<0.081	<0.081	<0.081	3.2	1.4	0.37	0.37
18	47.81	Benzo[b]fluoranthene	-	<0.098	-	6.6	3.6	0.99	0.71
19	47.85	Benzo[k]fluoranthene	-	-	-	-	-	-	-
20	49.1	Benzo[a]pyrene	-	-	-	1.6	0.83	0.21	<0.18
Oxy-PAH									
21	22.63	1.4-Naphthoquinone	-	-	-	<0.035	-	-	-
22	25.12	1-Naphthol	-	-	-	1.5	0.30	0.13	0.41
23	32.76	Xanthone	<0.008	<0.008		0.065	0.048	0.014	0.013
24	34.54	Anthrone	-	-	-	11	9.5	2.1	2.4
25	34.94	Anthraquinone	-	-	-	5.7	0.29	0.14	0.16
26	36.26	1.8-Naphthalic anhydride	<1.1	<1.1	<1.1	7.5	1.2	-	<1.1
Phthalates									
27	23.81	Dimethylphthalate	92	22	10	3.8	11	1.9	0.63
28	27.16	Diethylphthalate	18	37	28	3	8.8	1.2	0.47
29	33.05	Di-*n*-butyl phthalate	5567	6115	14,462	389	971	211	45
30	34.90	Benzyl butyl phthalate	911	892	1734	33	72	10	4.6
31	42.55	Bis(2-ethylhexyl)adipate	263	178	715	109	491	14	-
32	44.86	Di-*n*-octyl phthalate	5866	926	5381	36	38	6.7	3.8

**Table 2:** Major compounds identified via non-targeted screening.

Tentative Compound	Rt, min	M^+^/[M − H]^+^ *m*/*z*	M^+^_theor_ *m*/*z*	Δ *m*/*z*	Formula	Score	Estimated Concentration, ng/m^3^ *						
							S1	S2	S3	W1	W2	W3	W4
		CHO-compounds											
2(3H)-Furanone, 5-methyl-	6.30	98.0363	98.0362	0.30	C_5_H_6_O_2_	95	5.5	8.0	2.5	1.1	1.0	0.40	0.29
2(5H)-Furanone, 5-methyl-	8.76	98.0363	98.0362	0.50	C_5_H_6_O_2_	97	6.1	10	2.8	1.2	-	-	-
2-Furanone, 2,5-dihydro-3,5-dimethyl	13.31	112.0518	112.0519	−0.38	C_6_H_8_O_2_	88	8.3	9.9	4.8	1.0	1.9	2.3	0.70
6-Methyl-3,5-heptadiene-2-one	14.10	122.0727	122.0726	0.32	C_8_H_12_O	96	6.4	12	2.2	1.8	2.5	3.2	0.87
trans-Carveol	14.90	152.1196	152.1196	−0.10	C_10_H_16_O	90	18.	18	7.5	0.69	4.1	2.1	0.79
		*N*-containing compounds											
Pyridine, 2,3,6-trimethyl-	10.43	121.0886	121.0886	−0.10	C_8_H_11_N	98	7.0	5.8	0.49	0.86	1.5	0.31	0.27
Caprolactam	18.35	113.0835	113.0835	−0.12	C_6_H_11_NO	89	204	40	44	4.0	14	17	3.4
Pyridine, 3-(1-methyl-2-pyrrolidinyl)-, (S)-	21.05	162.1153	162.1152	0.43	C_10_H_14_N_2_	97	28	34	0.56	4.0	45	44	2.6
Diethyltoluamide	26.78	190.1225	190.1226	−0.54	C_12_H_17_NO	76	12	20	244	1.5	3.8	0.95	4.1
Benzenesulfonamide, *N*-butyl-	31.35	213.0820	213.0823	1.1	C_10_H_15_NO_2_S	76	29	115	182	2.3	3.9	29	10
		Phenols and monoaromatic compounds											
Benzene, 1-methoxy-4-methyl-	8.57	122.0726	122.0726	−0.41	C_8_H_10_O	97	23	37	14	2.1	-	-	-
p-Cymene	11.52	134.1090	134.1090	−0.37	C_10_H_14_	97	11	12	13	13	6.8	17	8.1
Phenol, 2-methyl-5-(1-methylethyl)-	17.81	150.1039	150.1039	−0.38	C_10_H_14_O	95	12	14	10	22	2.4	1.8	3.3
Vanillin	22.54	151.0391	151.0390	0.38	C_8_H_8_O_3_	92	15	11	10	132	59	2.1	0.72
2-Propanone, 1-(4-hydroxy-3-methoxyphenyl)-	25.55	180.0780	180.0781	−0.58	C_10_H_12_O_3_	93	0.53	0.30	1.1	8.3	1.0	0.001	0.001
		Oxy-PAHs											
Hydroxybiphenyl isomer	27.92	170.0727	170.0726	0.31	C_12_H_10_O	95	-	-	-	1.5	0.63	0.10	0.51
7-Methylnaphthalen-2-ol	28.08	158.0728	158.0726	1.0	C_11_H_10_O	97	-	-	-	1.2	0.28	0.74	0.08
1(2H)-Acenaphthylenone	28.37	168.0568	168.0570	−0.84	C_12_H_8_O	97	-	-	-	17	3.4	0.45	0.88
9H-Fluoren-9-one	30.15	180.0568	180.0570	−0.92	C_13_H_8_O	97	0.11	0.11	-	3.5	1.3	0.62	0.20
Cyclopenta(def)phenanthrenone	36.23	204.0569	204.0570	−0.36	C_15_H_8_O	96	-	0.02	-	0.81	0.23	0.09	0.13
		PAHs											
Acephenanthrylene	36.88	202.0772	202.0777	−2.5	C_16_H_10_	97	-	-	-	2.1	0.36	0.09	0.07
7H-Benzanthrene	39.02	215.0853	215.0855	−0.89	C_17_H_12_	91	-	-	-	0.74	0.38	0.07	0.07
Retene	39.28	234.1403	234.1403	−0.20	C_18_H_18_	97	-	-	-	1.2	1.1	0.08	0.08
Cyclopenta[cd]pyrene	42.10	226.0773	226.0777	−1.4	C_18_H_10_	96	-	-	-	2.6	0.56	0.13	0.36
Benzo[e]pyrene	48.88	252.0932	252.0934	−0.68	C_20_H_12_	97	0.008	-	-	0.91	0.19	0.03	0.04

*-All values in this table are semi-quantitative estimates based on peak areas relative to the nearest structural analog.

## Data Availability

The data presented in this study are available in the article and Supplementary Materials.

## Supplementary Materials

The following supporting information can be downloaded at https://www.mdpi.com/article/10.3390/molecules31101636/s1. Table S1: Target SVOCs, corresponding quantifier ions and limits of detection and quantification; Table S2: Diagnostic ratios in winter samples (W1–W4) for source identification; Table S3: Full list of tentative identified compounds via non-target screening and their semi-quantitative concentrations (ng/m^3^); Table S4. Meteorological data (temperature, RH, wind speed, wind direction) corresponding to the winter (W1–W4) and summer (S1–S3) sampling. Figure S1. Zoomed view of the extracted ion chromatograms (XIC) for the 37.4–37.7 min region of sample W1. The chromatograms were extracted with a mass tolerance of ±5 ppm. The figure illustrates the sepa-ration of co-eluting PAHs based on their characteristic exact masses (*m*/*z* 206.0722 (C15H10O), 204.0930 (C16H12), 218.0725 (C17H14)). Figure S2. TIC chromatograms. A standard mixture of target analytes with a concentration of 1 mg/L (black). An empty filter is analyzed immediately after the standard (red). No significant peaks were detect-ed in the blank (>0.1% of the standard signal). Table S5. Relative standard deviations (RSD) for 72 target compounds after three repetitions of thermal desorption from the filter (5 ng of each compound).
