# Supplementary material for: Targeted and Non-Targeted Screening of Organic Pollutants in Atmospheric Aerosols of Arctic Urban Agglomeration Using TD-GC-Orbitrap MS
Source: Molecules. 2026 May 13;31(10):1636. doi: 10.3390/molecules31101636 (PMC13209828; doi:10.3390/molecules31101636)
Supplement: Supplementary file 1 [file molecules-31-01636-s001.zip › molecules-4278040-supplementary.pdf]

Supplementary material

Targeted and Non-targeted Screening of Organic Pollutants in Atmospheric Aerosols of Arctic Urban Agglomeration by TD-GC-Orbitrap MS

Irina S. Shavrina[1], Kirill O. Sukhanov[1], Nikolay V. Ul'yanovskii[1], Dmitry S. Kosyakov [1]*, Albert T. Lebedev[2]

[1]Laboratory of Environmental Analytical Chemistry, Core Facility Center "Arktika", Northern (Arctic) Federal University, 163002 Arkhangelsk, Russia; i.shavrina@narfu.ru (I.S.S.); n.ulyanovsky@narfu.ru (N.V.U.); suhanov.k.o@edu.narfu.ru (K.O.S);
[2] Department of Materials Science, MSU-BIT University, Shenzhen 517182, China; a.lebedev@org.chem.msu.ru (A.T.L)
* Correspondence d.kosyakov@narfu.ru (D.S.K.)

Table S1. Target SVOCs, corresponding quantifier ions and limits of detection and quantification

№	tr, min	Compounds	CAS #	Formula	*m/z*	*LOD*	*LOD**	*LOQ**
						ng/filter	ng/m^3	
		**N-compounds**						
1	3.40	N-Nitrosodimethylamine	62–75–9	C2H6N2O	74.0474	0.34	0.18	0.6
2	3.34	Pyridine	110–86–1	C5H5N	79.0416	0.07	0.039	0.13
3	10.05	Aniline	62–53–3	C6H5NH2	93.0573	0.49	0.27	0.89
4	13.15	N-Nitroso-di-n-propylamine	621–64–7	C6H14N2O	113.1073	0.23	0.13	0.43
5	14.90	2-Nitrophenol	88–75–5	C6H5NO3	139.0263	0.51	0.28	0.93
6	17.04	4-Chloroaniline	106–47–8	C6H4NH2Cl	127.0183	0.1	0.056	0.19
7	22.56	2-Nitroaniline	88–74–4	C6H6N2O2	138.0423	0.24	0.13	0.44
8	23.14	1,4-Dinitrobenzene	100–25–4	C6H4N2O4	168.0164	0.52	0.28	0.95
9	23.64	1,3-Dinitrobenzene	99–65–0	C6H4N2O4	168.0164	0.32	0.18	0.59
10	23.97	2,6-Dinitrotoluene	606–20–2	C7H6N2O4	165.0294	0.023	0.013	0.04
11	24.18	1,2-Dinitrobenzene	528–29–0	C6H4N2O4	168.0164	1.1	0.65	2.1
12	24.72	3-Nitroaniline	99–09–2	C6H6N2O2	138.0423	3.5	1.9	6.4
13	25.70	2,4-Dinitrotoluene	121–14–2	C7H6N2O4	165.0294	0.059	0.032	0.11
14	26.80	4-Nitroaniline	100–01–6	C6H6N2O2	138.0423	11	6.0	20
15	27.70	Diphenylamine	122–39–4	C12H11N	169.0886	0.009	0.005	0.020
16	27.74	Azobenzene	103–33–3	C12H10N2	182.0838	0.039	0.022	0.07
17	32.33	Carbazole	86–74–8	C12H9N	167.0727	0.069	0.006	0.021
		**Phenols, benzyl alcohol**						
18	10.28	Phenol	108–95–2	C6H6O	94.0413	0.048	0.027	0.090
19	11.89	Benzyl alcohol	100–51–6	C7H8O	79.0542	0.12	0.069	0.23
20	12.62	2-Methylphenol	95–48–7	C7H8O	108.0569	0.13	0.076	0.25
21	13.29	3+4-Methylphenol	108–39–4	C7H8O	108.0569	0.27	0.15	0.50

22			106–44–5	C7H8O	108.0569	0.27	0.15	0.50
23	14.57	Isophorone	78–59–1	C9H14O	82.0412	0.029	0.016	0.050
24	15.45	2,4-Dimethylphenol	105–67–9	C8H10O	122.0726	0.045	0.025	0.080
				Halogenated SVOCs				
25	10.37	2-Chlorophenol	95–57–8	C6H5OCl	128.0023	0.045	0.024	0.080
26	10.4	Bis(2-chloroethyl)ether	111–44–4	C4H8Cl2O	62.9996	0.083	0.046	0.15
27	13.03	Hexachloroethane	67–72–1	C2Cl6	200.8407	0.2	0.10	0.34
28	15.84	Bis(2-chloroethoxy)methane	111–91–1	C5H10Cl2O2	93.0102	0.13	0.074	0.25
29	15.96	2,4-Dichlorophenol	120–83–2	C6H4Cl2O	161.9632	0.03	0.016	0.053
30	19.49	4-Chloro-3-methylphenol	59–50–7	C7H7ClO	142.0179	0.05	0.028	0.093
31	21.12	2,4,6-Trichlorophenol	95–95–4	C6H3Cl3O	195.9243	0.029	0.016	0.053
32	21.27	2,4,5-Trichlorophenol	88–06–2	C6H3Cl3O	195.9243	0.041	0.023	0.076
33	21.69	2-Chloronaphthalene	91–58–7	C10H7Cl	162.0229	0.013	0.007	0.023
34	25.95	2,3,5,6-Tetrachlorophenol	935–95–5	C6H2Cl4O	231.8822	0.27	0.15	0.50
35	26.15	2,3,4,6-Tetrachlorophenol	58–90–2	C6H2Cl4O	231.8822	0.19	0.10	0.34
36	27.06	4-Chlorophenyl phenyl ether	7005–72–3	C12H9ClO	204.0335	0.011	0.006	0.021
37	29.25	4-Bromophenyl phenyl ether	101–55–3	C12H9BrO	247.9828	0.009	0.005	0.017
38	29.65	Hexachlorobenzene	118–74–1	C6Cl6	283.8093	0.007	0.004	0.014
39	30.76	Pentachlorophenol	87–86–5	C6HCl5O	265.8432	1.7	0.97	3.2
				Polycyclic aromatic hydrocarbons				
40	16.41	Naphthalene	91–20–3	C10H8	128.062	0.027	0.015	0.049
41	19.55	2-Methylnaphthalene	91–57–6	C11H10	141.0699	0.010	0.006	0.018
42	20.01	1-Methylnaphthalene	90–12–0	C11H10	141.0699	0.010	0.005	0.018
43	23.55	Acenaphthylene	208–96–8	C12H8	152.0619	0.010	0.006	0.019
44	24.44	Acenaphthene	83–32–9	C12H10	153.0698	0.006	0.003	0.011
45	25.2	Dibenzofuran	53–70–3	C12H8O	168.0568	0.007	0.004	0.012
46	26.76	Fluorene	86–73–7	C13H10	165.0697	0.004	0.002	0.008
47	30.95	Phenanthrene	85–01–8	C14H10	178.0773	0.003	0.001	0.005
48	31.27	Anthracene	120–12–7	C14H10	178.0773	0.003	0.002	0.005
49	36.45	Fluoranthene	206–44–0	C16H10	202.0772	0.008	0.004	0.014
50	37.39	Pyrene	129–00–0	C16H10	202.0772	0.008	0.004	0.014
51	43.06	Benz[a]anthracene	56–55–3	C18H12	228.0931	0.055	0.03	0.10
52	43.22	Chrysene	218–01–9	C18H12	228.0931	0.044	0.024	0.081
53	47.81	Benzo[b]fluoranthene	205–99–2	C20H12	252.0929	0.053	0.029	0.098
54	47.85	Benzo[k]fluoranthene	207–08–9	C20H12	252.0929	0.044	0.024	0.081
55	49.1	Benzo[a]pyrene	50–32–8	C20H12	252.0929	0.096	0.053	0.18
56	53.95	Imdeno(1,2,3-cd)pyrene	193-93-5	C22H12	276.0929	1.5	0.84	2.7
57	54.42	Dibenz(a,h)anthracene	53-70-3	C22H14	278	2.1	1.1	3.8
58	54.54	Benzo[g,h,i]perylene	191–24–2	C22H12	276.0929	1.0	0.58	1.9
				Phthalates				
59	23.81	Dimethylphthalate	131–11–3	C10H10O4	163.0388	0.051	0.092	0.31
60	27.16	Diethylphthalate	84–66–2	C12H14O4	149.0232	0.022	0.039	0.13

61	33.05	Di-*n*-butyl phthalate	84–74–2	$C_{16}H_{22}O_4$	149.0232	0.33	0.6	2
62	34.9	Benzyl butyl phthalate	85–68–7	$C_{19}H_{20}O_4$	149.0232	0.086	0.15	0.51
63	42.55	Bis(2-ethylhexyl)adipate	103–23–1	$C_{22}H_{42}O_4$	129.0545	2.2	4	13
64	44.86	Di-*n*-octyl phthalate	117–84–0	$C_{24}H_{38}O_4$	149.0232	0.42	0.75	2.5
				Oxy, Nitro-PAH				
65	22.63	1.4-Naphthoquinone	130-15-4	$C_{10}H_6O_2$	158.0362	0.76	0.42	1.4
66	25.12	1-Naphthol	90-15-3	$C_{10}H_8O$	144.05697	0.048	0.085	0.28
67	28.21	2-Nitronaphthalene	581-89-5	$C_{10}H_7NO_2$	173.04713	0.035	0.019	0.065
68	32.76	Xanthone	90-47-1	$C_{13}H_8O_2$	196.0518	0.004	0.002	0.008
69	32.99	Acenaphthenequinone	82-86-0	$C_{12}H_6O_2$	182.0362	0.022	0.39	1.3
70	34.54	Anthrone	90-44-8	$C_{14}H_{10}O$	194.0726	0.77	1.4	4.5
71	34.94	Anthraquinone	84-65-1	$C_{14}H_8O_2$	208.0518	0.013	0.025	0.083
72	36.26	1.8-Naphthalic anhydride	81-84-5	$C_{12}H_6O_3$	198.03115	0.18	0.33	1.1

* - recalculated for air sample volume of 1.8m^3

Table S2 – Diagnostic ratios in winter samples (W1-W4) for source identification

Diagnostic Ratio	W1	W2	W3	W4	Source
Ant/(Ant+Phe)	0.12	0.17	0.14	0.16	<0.1 Petrogenic<br>**>0.1 Pyrogenic**
Fla/(Fla+Pyr)	0.49	0.48	0.45	0.5	<0.4 Petrogenic<br>**0.4–0.5 Fossil fuel combustion**<br>>0.5 Grass, wood, coal combustion
BaA/(BaA+Chr)	0.55	0.56	0.42	0.49	<0.2 Petrogenic<br>**>0.35 Combustion (Pyrogenic)**

Table S3. Full list of tentative identified compounds by non-target screening and their semi-quantitative concentrations (ng/m^3)

	Compounds	Rt	m/z	Formula	Reference compound for semi-quantification	Score	S1	S2	S3	W1	W2	W3	W4
	**CHO-compounds**												
1	Furan, 2-ethyl-5-methyl-	3.17	95.0492	C7H10O	Phenol	97	1.2	1.4	0.93	-	-	-	-
2	3,4-dimethylfuran	3.29	96.0570	C6H8O	Phenol	97	0.37	0.54	0.48	0.03	-	-	-
3	2-Propenoic acid, 2-methyl-	4.93	86.0362	C4H6O2	Phenol	93	0.41	1.0	0.001	1.3	0.55	0.21	0.20
4	5-Hepten-2-one, 6-methyl-	5.64	126.1040	C8H14O	Phenol	92	0.56	0.48	0.16	-	-	-	-
5	6-Methyl-3,5-heptadiene-2-one	5.92	109.0649	C8H12O	Phenol	97	1.1	1.3	0.76	0.20	-	-	-
6	2(3H)-Furanone, 5-methyl-	6.30	98.0363	C5H6O2	Phenol	95	5.4	8.0	2.5	1.1	1.07	0.40	0.29
7	Ethanone, 1-(3-methylenecyclopentyl)-	6.88	81.0699	C8H12O	Phenol	96	3.4	5.2	1.4	1.5	1.2	0.69	0.54
8	3,3-Dimethyl-6-methylenecyclohexene	7.06	107.0856	C9H14	Phenol	93	2.1	2.9	1.3	0.51	0.16	0.43	0.49
9	4,4-Dimethyl-2-cyclopenten-1-one	7.13	95.0492	C7H10O	Phenol	98	0.80	0.90	0.35	0.03	-	-	-
10	2-Butenoic acid, 2-methyl-, (Z)-	8.18	82.0413	C5H8O2	Phenol	92	2.4	2.4	0.82	0.19	-	-	-
11	2-Cyclopenten-1-one, 3,4-dimethyl-	8.47	110.0728	C7H10O	Phenol	92	0.63	0.21	0.19	-	-	-	-
12	Cyclohexanone, 2-acetyl-	8.63	97.0648	C8H12O2	Phenol	75	1.7	2.0	0.57	0.13	-	-	-
13	2(5H)-Furanone, 5-methyl-	8.76	55.0180	C5H6O2	Phenol	97	6.1	10	2.8	1.1	-	-	-
14	2-Acetylcyclopentanone	8.79	83.0491	C7H10O2	Phenol	97	0.65	0.71	0.23	0.10	-	-	-
15	2(5H)-Furanone, 5,5-dimethyl-	9.20	69.0335	C6H8O2	Phenol	95	8.4	9.8	3.5	-	-	-	-
16	2(5H)-Furanone, 5,5-dimethyl-	9.46	97.0285	C6H8O2	Phenol	90	1.5	1.7	0.97	0.46	-	-	-
17	1,3,5-Cycloheptatriene, 3,7,7-trimethyl-	9.71	119.0855	C10H14	Phenol	90	0.79	0.80	0.73	0.75	0.60	1.53	0.71
18	2H-Pyran-2-methanol, 3,4-dihydro-2,5-dimethyl-	11.33	142.0989	C8H14O2	Phenol	91	2.4	1.7	0.76	0.50	0.98	0.09	0.14
19	1,4-Cyclohex-2-enedione	11.36	110.0363	C6H6O2	Phenol	93	4.4	5.3	3.0	0.82	1.53	1.19	0.48
20	2-Butylcyclopenanone	12.63	84.0571	C9H16O	Phenol	95	1.4	1.2	0.19	0.04	-	-	-
21	2-Furanone, 2,5-dihydro-3,5-dimethyl	13.31	69.0335	C6H8O2	Phenol	88	8.2	9.9	4.8	1.0	1.8	2.3	0.71
22	Cyclohexanecarboxylic acid, ethenyl ester	13.88	83.0491	C11H20O3	Phenol	74	8.4	8.3	4.9	0.14	-	-	-
23	6-Methyl-3,5-heptadiene-2-one	14.10	109.0649	C8H12O	Phenol	96	6.4	12	2.2	1.8	2.5	3.1	0.87
24	Maltol	14.39	126.0312	C6H6O3	Phenol	84	0.59	0.41	0.58	-	-	-	-
25	2H-Pyran-2-one, 4,6-dimethyl-	14.53	96.0570	C7H8O2	Phenol	86	0.54	0.77	0.65	-	-	-	-
26	1,3-Cyclohexadiene, 5,6-dimethyl-	14.71	93.0699	C8H12	Phenol	93	0.56	0.48	0.55	2.0	1.6	5.6	3.3

27	trans-Carveol	14.90	152.1201	C10H16O	Phenol	90	18.	18.	7.5	0.69	4.0	2.1	0.79
28	Bicyclo[3.1.1]heptan-2-one, 6,6-dimethyl-, (1R)-	15.03	83.0491	C9H14O	Phenol	72	0.87	0.77	2.08	0.21	1.3	0.78	0.44
29	2-Cyclohexen-1-one, 4,4-dimethyl-	15.10	82.0777	C8H12O	Phenol	90	1.2	1.3	1.2	0.28	-	-	-
30	2-Cyclopenten-1-one, 3-methoxy-5-methyl-	15.32	126.0676	C7H10O2	Phenol	94	1.3	1.6	2.07	0.06	-	-	-
31	Ethanone, 1-(1,4-dimethyl-3-cyclohexen-1-yl)-/Pulegone	16.21	109.1013	C10H16O	Naphthalene	89	2.2	2.1	0.61	0.80	1.1	4.7	1.3
32	1,4:3,6-Dianhydro-α-d-glucopyranose	17.16	69.0334	C6H8O4	Naphthalene	96	-	-	-	2.1	1.1	-	-
33	Benzofuran, 4,7-dimethyl-	17.29	145.0648	C10H10O	Naphthalene	95	2.3	2.8	1.4	1.1	-	8.4	4.7
34	1-Propanone, 1-(2-furanyl)-	17.80	95.0492	C7H8O2	Naphthalene	94	9.6	11	4.1	0.41	-	-	-
35	1-Methylindan-2-one	20.13	131.0492	C10H10O	Naphthalene	96	0.65	0.74	0.61	4.4	-	-	-
36	2(3H)-Benzofuranone, 3-methyl-	20.82	148.0520	C9H8O2	Naphthalene	84	0.32	0.37	0.82	1.0	0.82	0.30	0.29
37	Furan, 2,2'-methylenebis-	21.16	148.0520	C9H8O2	Naphthalene	89	0.40	0.47	0.40	0.30	0.44	0.16	0.13
38	4-pentylphenyl 4-propylbenzoate	21.29	147.0441	C21H26O2	Naphthalene	80	1.6	1.6	0.49	0.71	1.1	0.64	0.61
39	3-Butylisobenzofuran-1(3H)-one	21.44	105.0336	C12H14O2	Naphthalene	90	1.5	1.4	-	1.2	7.5	9.7	5.4
40	Maltol	21.70	126.0676	C6H6O3	Naphthalene	90	0.26	0.24	0.14	-	-	-	-
41	1,3-Isobenzofurandione, 4-methyl-	22.55	134.0363	C9H6O3	Naphthalene	88	1.7	1.3	0.56	0.89	0.59	0.22	0.32
42	Ethanone, 1-(2,3-dihydro-1H-inden-5-yl)-	23.29	145.0648	C11H12O	Naphthalene	94	0.70	0.58	0.29	3.2	2.1	79	4.4
43	cis-Calamenene	25.43	159.1168	C15H22	Naphthalene	69	0.65	1.1	0.37	1.9	1.1	1.4	0.50
44	2(4H)-Benzofuranone, 5,6,7,7a-tetrahydro-4,4,7a-trimethyl-, (R)-	25.60	111.0441	C11H16O2	Naphthalene	94	0.71	1.26	0.88	-	-	0.20	0.07
45	Octanoic acid, 2-ethylhexyl ester	27.82	127.0390	C16H32O2	Naphthalene	83	2.2	2.3	2.2	-	-	-	-
46	Cyclopentaneacetic acid, 3-oxo-2-pentyl-, methyl ester	28.45	83.0491	C13H22O3	Naphthalene	96	10	12	6.8	-	5.2	0.47	1.1
47	Ambrox	30.68	221.1898	C16H28O	Naphthalene	74	1.7	2.3	0.37	-	1.3	0.42	0.21
48	Hexadecanoic acid, methyl ester	34.14	87.0441	C17H34O2	Anthracene	94	1.9	5.4	4.0	0.12	1.5	0.31	0.95
49	4b,8-Dimethyl-2-isopropylphenanthrene, 4b,5,6,7,8,8a,9,10-octahydro-	35.09	159.1167	C19H28	Anthracene	91	-	-	-	0.09	0.10	0.01	0.02
50	1H-Naphtho[2,1-b]pyran, 3-ethenyldodecahydro-3,4a,7,7,10a-pentamethyl-, [3S-(3α,4aα,6aβ,10aα,10bβ)]-	35.40	257.2262	C20H34O	Anthracene	68	0.01	0.01	0.01	0.02	0.01	0.01	0.01
51	1H-Naphtho[2,1-b]pyran, 3-ethenyldodecahydro-3,4a,7,7,10a-pentamethyl-, [3R-(3α,4aβ,6aα,10aβ,10bα)]-	35.81	257.2262	C20H34O	Anthracene	91	0.04	0.05	0.02	0.04	0.04	0.02	0.01

52	Methyl dehydroabietate	35.27	239.1792	C21H30O2	Anthracene	85	-	-	-	0.17	0.26	0.04	0.03
53	4b,8-Dimethyl-2-isopropylphenanthrene, 4b,5,6,7,8,8a,9,10-octahydro-	35.79	159.1167	C19H28	Anthracene	92	-	-	-	0.20	0.30	0.04	0.06
54	10,18-Bisnorabieta-5,7,9(10),11,13-pentaene	37.22	223.1481	C18H22	Anthracene	94	-	-	0.00	0.16	0.24	0.02	-
55	Benzene, 1,1'-(4,4-dimethyl-1-butene-1,4-diyl)bis-	37.33	221.1323	C18H20	Anthracene	94	-	-	-	0.10	0.13	0.01	0.03
56	Methyl dehydroabietate	41.46	239.1792	C21H30O2	Anthracene	97	0.12	0.12	0.14	1.8	3.0	0.28	0.24
57	1-Phenanthrenecarboxylic acid, 1,2,3,4,4a,9,10,10a-octahydro-1,4a-dimethyl-7-(1-methylethyl)-9-oxo-, methyl ester	45.17	253.1585	C21H28O3	Anthracene	96	-	-	-	0.26	0.30	0.00	0.00
						**Sum**	**132**	**162**	**73**	**36**	**46**	**125**	**30**
	**N-containing SVOC**												
1	Pyridine, 2-methyl-	4.90	93.0573	C6H7N	Pyridine	99	1.1	1.1	0.35	0.71	0.53	0.15	0.30
2	1H-Pyrrole, 3-methyl-	5.51	80.0495	C5H7N	Pyridine	97	2.9	5.2	1.0	7.1	5.1	0.69	3.1
3	Pyridine, 3/4-methyl-	6.05	93.0574	C6H7N	Pyridine	95	3.6	3.4	1.0	2.3	1.4	0.91	0.50
4	N-Vinylimidazole	6.47	94.0526	C5H6N2	Pyridine	93	1.2	0.85	0.36	0.18	-	-	-
5	Pyridine, 2,5-dimethyl- (isomer)	6.79	107.0730	C7H9N	Pyridine	95	1.7	1.5	0.10	0.78	0.49	0.04	0.08
6	Pyridine, 3,5-dimethyl-(isomer)	8.31	107.0729	C7H9N	Pyridine	98	1.7	1.8	0.38	1.7	1.5	0.26	0.40
7	Pyridine, 2,5-dimethyl- (isomer)	8.77	107.0729	C7H9N	Pyridine	98	0.56	0.57	0.17	0.88	0.58	0.09	0.10
8	1H-Imidazole, 4-methyl-	9.02	82.0525	C4H6N2	Pyridine	82	0.37	0.49	0.22	1.0	0.44	0.45	0.30
9	Pyridine, 3-ethyl-	9.28	106.0652	C7H9N	Pyridine	85	0.43	0.36	0.13	0.44	0.51	0.16	0.09
10	Pyridine, 2,3-dimethyl- (isomer)	9.91	107.0730	C7H9N	Pyridine	96	0.54	0.52	0.14	0.47	0.40	-	-
11	Pyridine, 2,3,6-trimethyl-	10.43	121.0886	C8H11N	Pyridine	98	7.0	5.8	0.49	0.86	1.5	0.31	0.27
12	Pyridine, 2,5-dimethyl- (isomer)	10.75	107.0729	C7H9N	Pyridine	96	1.0	0.96	0.33	0.49	0.50	0.17	0.10
13	Pyridine, 2,3,6-trimethyl-	12.48	121.0886	C8H11N	Pyridine	93	5.9	6.1	0.70	0.77	3.7	0.62	0.40
14	5H-1-Pyrindine, 6,7-dihydro-	12.89	118.0651	C8H9N	Pyridine	96	0.94	0.96	0.34	4.0	0.71	0.13	0.15
15	4(1H)-Pyridone	13.49	95.0366	C5H5NO	Pyridine	98	4.3	3.9	1.2	1.2	1.6	1.1	0.25
16	Pyridine, 4-(1,1-dimethylethyl)-	13.53	120.0808	C9H13N	Pyridine	95	3.0	2.8	0.49	2.0	1.2	0.44	0.49
17	Ethanone, 1-(3-pyridinyl)-	13.71	106.0288	C7H7NO	Pyridine	95	0.67	0.58	0.55	0.30	0.21	0.04	0.05
18	Ethanone, 1-(3-pyridinyl)-	14.14	106.0289	C7H7NO	Pyridine	95	0.91	0.66	0.15	0.22	0.43	0.30	0.09
19	4(1H)-Pyridone	14.48	95.0366	C5H5NO	Pyridine	89	-	-	-	4.0	-	-	-

20	Pyridine, 2-methyl-5-(1-methylethenyl)-	14.93	133.0887	C9H11N	Pyridine	95	2.1	2.3	0.36	0.60	0.45	0.14	0.36
21	Phenol, 4-amino-	14.96	109.0522	C6H7NO	Phenol	84	0.72	0.72	0.23	0.84	1.06	0.35	0.20
22	1-Piperidinecarboxaldehyde	15.03	113.0835	C6H11NO	Pyridine	95	0.50	0.30	0.36	1.00	0.83	3.07	0.39
23	Methyl nicotinate	15.08	136.0395	C7H7NO2	Pyridine	92	0.11	0.07	0.02	0.06	-	-	-
24	1H-Indole, 2,3-dihydro-	15.56	118.0651	C8H9N	Phenol	95	1.7	2.1	0.33	0.50	-	-	-
25	Acetophenone, 4'-amino-	16.59	120.0444	C8H9NO	Phenol	90	1.4	1.3	0.44	0.76	-	-	-
26	Quinoline	17.88	129.0571	C9H7N	Carbazole	97	-	-	-	11	4.2	0.61	1.6
27	Caprolactam	18.35	85.0522	C6H11NO	Carbazole	89	204	40	44	4.0	14	17	3.4
28	Isoquinoline	18.55	129.0574	C9H7N	Carbazole	96	7.4	5.7	0.43	23	11	2.3	5.8
29	Quinoline, methyl- isomer	19.85	143.0729	C10H9N	Carbazole	86	0.54	0.52	0.12	2.0	-	-	-
30	Quinoline, methyl- isomer	20.42	143.0729	C10H9N	Carbazole	89	1.1	1.3	0.08	3.4	1.7	0.43	0.63
31	Isoquinoline, 1-methyl-	20.81	143.0730	C10H9N	Carbazole	95	2.1	2.0	0.12	5.1	2.9	0.86	0.85
32	Pyridine, 3-(1-methyl-2-pyrrolidinyl)-, (S)-	21.05	84.0808	C10H14N2	Pyridine	97	28	34	0.56	4.08	45	44	2.6
33	Quinoline, methyl- isomer	21.51	143.0729	C10H9N	Carbazole	89	0.12	0.13	0.13	0.40	-	-	-
34	Quinoline, methyl- isomer	21.66	143.0729	C10H9N	Carbazole	96	0.77	0.75	0.13	2.4	1.2	0.47	0.43
35	Quinoline, methyl- isomer	21.73	144.0729	C10H9N	Carbazole	85	0.53	0.56	0.08	1.0	1.5	0.50	0.40
36	Quinoline, methyl- isomer	21.93	144.0729	C10H9N	Carbazole	94	0.49	0.06	0.09	2.1	1.4	0.64	0.88
37	Quinoline, methyl- isomer	22.05	144.0729	C10H9N	Carbazole	91	0.36	0.40	-	2.6	1.8	0.88	0.81
38	1,3,5-Triazine-2,4,6(1H,3H,5H)-trione, 1,3,5-tri-methyl-	21.87	171.0638	C6H9N3O3	Carbazole	97	-	-	-	3.7	0.28	0.06	0.06
39	Quinoline, 2,6-dimethyl-	22.35	157.0886	C11H11N	Carbazole	94	0.21	0.21	0.04	1.4	0.87	0.13	0.20
40	1H-Isoindole-1,3(2H)-dione, 2-methyl-	22.84	161.0470	C9H7NO2	Carbazole	97	3.6	1.1	0.31	5.9	0.68	0.18	0.16
41	Pyridine, 3-(3,4-dihydro-2H-pyrrol-5-yl)-	22.94	145.0760	C9H10N2	Pyridine	91	2.5	2.8	0.21	0.59	3.34	5.04	0.36
42	Quinoline, dimethyl- isomer	23.34	157.0886	C11H11N	Carbazole	86	4.5	1.4	1.7	1.9	1.4	0.75	0.33
43	Quinoline, dimethyl- isomer	23.42	157.0886	C11H11N	Carbazole	91	0.00	0.15	0.00	0.83	0.52	0.24	0.18
44	Quinoline, dimethyl- isomer	23.75	157.0886	C11H11N	Carbazole	98	-	-	-	1.6	1.3	0.52	0.40
45	Pyridine, 2-phenyl-	23.80	155.0730	C11H9N	Pyridine	85	0.93	0.95	0.40	0.82	0.84	0.60	0.16
46	Pyridine, 3-phenyl-	23.97	155.0730	C11H9N	Pyridine	92	0.46	0.56	0.40	1.0	0.58	0.36	0.13
47	Phthalimide	24.16	103.0417	C8H5NO2	Carbazole	95	8.8	6.1	2.4	3.3	1.4	0.49	-
48	Nicotyrine	24.37	158.0839	C10H10N2	Pyridine	94	2.4	3.0	0.06	1.2	12	2.0	24
49	Naphthalene, 1-isocyano-	24.60	153.0572	C11H7N	Carbazole	89	-	-	-	2.5	0.56	0.28	0.39

50	Quinoline, 5,8-dimethyl-	25.01	157.0886	C11H11N	Carbazole	87	-	-	-	0.62	0.55	0.25	-
51	2-Naphthalenecarbonitrile	25.23	153.0572	C11H7N	Carbazole	91	-	-	-	3.3	0.66	0.18	0.42
52	4,4'-Bipyridine	25.68	156.0684	C10H8N2	Pyridine	91	0.66	0.96	0.10	0.28	1.1	1.6	0.11
53	4-Nitroguaiacol	25.82	169.0369	C7H7NO4	2-Nitrophenol	84	0.05	0.05	0.00	1.5	0.24	0.001	0.001
54	Benz[cd]indol-2(1H)-one	25.87	169.0521	C11H7NO	Carbazole	82	-	-	-	0.83	0.33	0.05	0.08
55	Diethyltoluamide	26.78	190.1225	C12H17NO	Diethylphthalate	86	12	20	244	1.5	3.8	0.95	4.1
56	2,6-di-tert-Butyl-4-(dimethylaminomethyl)phenol	29.47	219.1744	C17H29NO	2-Nitrophenol	81	1.3	0.45	0.34	0.55	5.1	0.87	-
57	Benzenesulfonamide, N-butyl-	31.35	141.0004	C10H15NO2S	2-Nitrophenol	91	29	115	182	2.3	3.9	30	10
58	5-acenaphthylenecarbonitrile	31.37	177.0573	C13H7N	Carbazole	88	-	-	-	3.0	0.59	-	0.45
59	Benzoquinoline isomer	31.40	179.0725	C13H9N	Carbazole	95	-	-	-	1.2	0.54	0.07	0.25
60	Benzoquinoline isomer	31.85	139.0502	C7H9NO2	Carbazole	93	0.70	0.73	0.001	-	-	-	-
61	Rolziracetam	31.85	139.0502	C7H9NO2	Carbazole	81	2.5	2.1	0.001	-	-	-	-
62	Pyrazolo[3,4-b]pyridin-6-one, 1,7-dihydro-1,3,4-trimethyl-	31.91	177.0897	C9H11N3O	Carbazole	93	-	-	-	2.0	-	-	-
63	Benzoquinoline isomer	32.52	179.0725	C13H9N	Carbazole	95	-	-	-	9.5	6.0	0.63	0.69
64	Benzoquinoline isomer	32.56	179.0725	C13H9N	Carbazole	95	-	-	-	13	0.001	0.001	0.001
65	Benzoquinoline isomer	32.74	179.0725	C13H9N	Carbazole	94	-	-	-	3.4	0.52	0.08	-
66	Phenol, 2,6-di-tert-butyl-4-nitro-	33.55	236.1280	C14H21NO3	Carbazole	85	11	2.1	0.70	1.2	4.7	-	-
67	Isoquinoline, 1-phenyl-	34.23	204.0809	C15H11N	Carbazole	69	-	-	-	1.3	0.52	-	-
68	Acridine, 9-methyl-	34.26	193.0887	C14H11N	Carbazole	97	-	-	-	1.5	0.61	0.18	-
69	Isoquinoline, 1-phenyl-	34.62	204.0809	C15H11N	Carbazole	88	-	-	-	0.68	0.30	-	-
70	9-Acridinecarboxylic acid	36.28	223.0628	C14H9NO2	Carbazole	84	0.18	0.24	0.001	0.001	0.001	0.001	0.001
71	Quinoline, 2-phenyl-	36.34	205.0884	C15H11N	Carbazole	96	0.001	0.001	0.001	3.08	1.43	0.10	0.08
72	2-Methyl-6-phenylpyrimidin-4-ol	36.57	117.0574	C11H10N2O	Carbazole	91	4.1	5.0	0.001	-	-	-	-
73	Drometrizole	36.67	225.0898	C13H11N3O	Carbazole	89	0.62	0.72	0.001	-	-	-	-
74	Anthracene, 9-nitro-	37.93	176.0621	C14H9NO2	Carbazole	92	-	-	-	0.83	-	-	-
75	Naphtho(2,1,8-def)quinoline	37.95	203.0729	C15H9N	Carbazole	95	-	-	-	0.04	0.01	0.001	0.001
76	9-Anthracenecarbonitrile	38.17	203.0729	C15H9N	Carbazole	95	-	-	-	3.5	0.50	0.15	0.20
						**Sum**	**379**	**296**	**489**	**177**	**166**	**124**	**69**

					Phenols and monoaromatics									
1	Benzene, 1-methoxy-2-methyl-	7.66	122.0726	C8H10O	Phenol	96	2.2	2.7	1.8	0.97	2.5	3.4	1.4
2	Benzene, 1-methoxy-4-methyl-	8.57	122.0726	C8H10O	Phenol	97	23	37	14	2.1	-	-	-
3	3-Methylbenzyl alcohol	8.85	122.0726	C8H10O	Phenol	94	1.9	2.3	1.0	0.43	2.5	3.4	1.3
4	Benzene, (1-methylethyl)-	9.66	105.0700	C9H12	Phenol	98	1.4	1.6	1.0	0.18	-	-	-
5	p-Cymene	11.52	119.0855	C10H14	Phenol	97	11	12	13	12	6.8	17	8.1
6	Benzene, (1-methylethyl)-	11.79	120.0934	C9H12	Phenol	92	3.5	5.3	2.8	1.3	0.69	2.6	1.6
7	4-Methoxy-2,6-dimethylphenol	12.77	137.0597	C9H12O2	Phenol	95	5.4	7.7	4.6	0.38	-	-	-
8	Benzene, 1,2,4-trimethyl-	12.87	120.0934	C9H12	Phenol	92	1.6	1.6	0.64	0.25	-	-	-
9	Benzaldehyde, 4-methyl-	12.90	119.0492	C8H8O	Phenol	96	12	9.9	7.2	6.6	-	-	-
10	Phenol, 3,5-diethyl-	13.01	121.1012	C10H14O	Phenol	88	1.2	1.2	0.43	0.66	-	-	-
11	Benzene, 1-methyl-3-(1-methylethenyl)-	13.59	132.0934	C10H12	Phenol	95	6.9	8.5	7.3	9.9	4.6	11	7.6
12	Benzene, 1,4-dimethoxy-2-methyl-	14.77	137.0597	C9H12O2	Phenol	92	2.0	0.64	0.42	-	-	-	-
13	4-Ethylcatechol	15.57	123.0441	C8H10O2	Phenol	86	2.4	3.4	1.5	0.92	-	-	-
14	Acetophenone, 4'-hydroxy-	15.78	121.0284	C8H8O2	Phenol	85	3.3	4.5	2.5	-	-	-	-
15	p-Cymen-7-ol	15.99	135.0804	C10H14O	Phenol	96	31	36	25	25	14	2	21
16	Phenol, 3-ethyl-	16.07	122.0726	C8H10O	Phenol	92	6.0	14	8.3	3.1	2.5	6.7	1.5
17	Benzaldehyde, 2,4-dimethyl-	16.16	133.0648	C9H10O	Phenol	80	1.2	1.3	1.4	0.92	0.37	1.0	0.90
18	Benzene, 1,2-dimethoxy-	16.41	138.0676	C8H10O2	Phenol	86	2.7	3.0	1.7	0.46	3.3	1.1	-
19	Benzene, 1-ethyl-4-methoxy-	17.01	121.0648	C9H12O	Phenol	96	2.1	1.4	1.1	3.2	8.6	1.6	1.1
20	Phenol, o-amino-	17.52	109.0524	C6H7NO	Phenol	87	4.0	0.57	0.001	0.001	-	-	-
21	1,3-Benzenediol, 4-ethyl-	17.67	123.0441	C8H10O2	Phenol	93	2.6	2.9	1.7	0.89	-	-	-
22	Phenol, 2-methyl-5-(1-methylethyl)-	17.81	135.0804	C10H14O	Phenol	95	12	14	10	21	2.3	1.8	3.3
23	Benzene, 1,3-bis(1-methylethenyl)-	19.12	158.1090	C12H14	Phenol	96	2.8	3.3	3.9	-	-	-	-
24	Benzaldehyde, 4-hydroxy-	20.02	121.0285	C7H6O2	Phenol	94	-	-	-	10.79	11.18	0.05	2.32
25	Benzaldehyde, 4-hydroxy-	21.51	121.0285	C9H10O2	Phenol	88	-	-	-	21	5.6	-	-
26	4H-1-Benzopyran-4-one	22.08	146.0362	C9H6O2	Phenol	86	0.53	0.002	0.10	5.2	2.6	0.63	1.2
27	Coumarin	22.30	118.0413	C9H6O2	Phenol	96	1.5	1.9	0.67	9.8	4.1	1.7	2.3
28	Vanillin	22.54	151.0391	C8H8O3	Phenol	92	15.37	11	10	132	59	2.1	0.72
29	m-Ethylacetophenone	22.38	133.0648	C10H12O	Phenol	91	2.5	3.5	5.0	5.2	1.8	4.1	4.6

30	1H-Indenol	22.81	132.0570	C9H8O	Phenol	92	0.64	0.86	-	4.9	1.2	1.0	0.44
31	Coumarin	23.37	118.0413	C9H6O2	Phenol	93	6.6	5.6	0.76	10	7.0	1.9	2.6
32	2,6-Di-tert-butyl-4-hydroxy-4-methylcyclohexa-2,5-dien-1-one	24.14	165.0908	C15H24O2	Phenol	92	181	20	16	3.5	5.5	0.63	0.93
33	Apocynin	24.47	151.0390	C9H10O3	Phenol	93	0.83	1.8	2.8	21	6.0	0.16	-
34	Menadione	25.12	172.0518	C11H8O2	Phenol	80	-	-	-	5.3	3.3	1.0	1.0
35	Ethanone, 1-(3-hydroxy-4-methoxyphenyl)-	25.52	151.0390	C8H8O3	Phenol	96	-	-	-	3.4	0.45	0.001	0.003
36	2-Propanone, 1-(4-hydroxy-3-methoxyphenyl)-	25.55	137.0597	C10H12O3	Phenol	93	0.53	0.30	1.1	8.3	1.0	0.002	0.002
37	Benzaldehyde, 4-hydroxy-3,5-dimethoxy-	28.51	182.0574	C9H10O4	Phenol	87	-	-	-	1.4	0.53	-	-
38	Benzoic acid, 2-ethylhexyl ester	29.60	105.0335	C15H22O	Phenol	89	160	84	11	3.3	13	1.2	1.6
39	Salicylic acid	31.69	120.0206	C7H6O3	Phenol	94	10	6.9	-	-	-	-	-
40	Benzoic acid, octyl ester	32.70	123.0441	C12H16O2	Phenol	75	14	15	-	-	-	-	-
41	Salicylic acid ester	33.30	120.0206	C7H6O3	Phenol	81	4.7	5.4	2.8	-	-	-	-
						Sum	545	336	166	340	173	87	66
	Oxy-PAH												
1	1,3-Naphthalenediol	23.92	160.0519	C10H8O2	1-Naphthol	86	0.38	0.49	0.32	0.33	0.29	0.11	0.17
2	1-Naphthalenecarboxaldehyde	24.80	156.0571	C11H8O	1-Naphthol	93	-	-	-	1.0	-	-	-
3	2-Naphthalenecarboxaldehyde	24.87	155.0495	C11H8O	1-Naphthol	90	-	-	-	3.7	0.88	0.38	1.0
4	2-Naphthalenol	25.24	144.0570	C10H8O	1-Naphthol	94	-	-	-	1.9	0.56	0.20	0.22
5	Ethanone, 1-(1-Naphthalenyl)-	27.39	155.0495	C12H10O	1-Naphthol	84	0.20	0.16	0.04	2.1	1.0	0.25	0.43
6	Hydroxybiphenyl isomer	27.58	169.0649	C12H10O	1-Naphthol	90	-	-	-	0.69	0.30	0.07	0.33
7	Hydroxybiphenyl isomer	27.70	169.0649	C12H10O	1-Naphthol	87	-	-	-	1.3	0.62	0.12	0.10
8	Hydroxybiphenyl isomer	27.92	170.0724	C12H10O	1-Naphthol	95	-	-	-	1.4	0.63	0.10	0.51
9	7-Methylnaphthalen-2-ol	28.08	158.0727	C11H10O	1-Naphthol	97	-	-	-	1.1	0.28	0.74	0.08
10	1(2H)-Acenaphthylenone	28.37	168.0568	C12H8O	Acenaphthylene	97	-	-	-	17	3.4	0.45	0.88
11	6H-Dibenzo[b,d]-pyran	29.12	182.0726	C13H10O	Acenaphthylene	91	0.00	0.11	0.00	0.87	1.6	0.82	0.57
12	p-Hydroxybiphenyl (isomer)	29.45	170.0724	C12H10O	1-Naphthol	94	-	-	-	2.8	0.84	0.05	0.08
13	p-Hydroxybiphenyl (isomer)	29.65	170.0724	C12H10O	1-Naphthol	92	-	-	-	2.2	0.65	0.06	-
14	9H-Fluoren-9-one	30.15	180.0568	C13H8O	Fluorene	97	0.11	0.11	-	3.5	1.3	0.60	0.20
15	2-Dibenzofuranol	30.66	184.0518	C12H8O2	Dibenzofuran	91	-	-	-	0.24	0.04	-	-
16	Benzyl Benzoate	30.84	105.0336	C14H12O2	Dibenzofuran	90	0.14	0.11	0.02	0.01	0.08	0.02	0.02

17	1H-Phenalen-1-one	31.42	180.0568	C13H8O	Phenanthrene	87	-	-	-	0.08	0.03	0.01	0.02
18	Dibenz[b,e]oxepin	31.87	194.0724	C14H10O	Phenanthrene	82	0.02	0.02	0.00	0.16	0.09	0.03	0.05
19	Benzo[b]benzofurancarboxaldehyde isomer	32.41	195.0442	C13H8O2	Phenanthrene	92	-	-	-	0.16	0.06	0.01	0.02
20	4-Phenanthrenol	32.61	194.0724	C14H10O	Phenanthrene	90	0.02	0.02	-	0.11	0.08	0.03	0.04
21	3-Phenyl-benzofuran	32.87	194.0724	C14H10O	Dibenzofuran	98	0.03	0.03	0.00	0.16	0.08	0.05	0.04
22	1H-Phenalen-1-one	33.41	152.0619	C13H8O	Phenanthrene	95	-	-	-	3.5	0.99	0.49	0.32
23	1H,3H-Naphtho[1,8-cd]pyran-1-one	33.81	155.0495	C12H8O2	Phenanthrene	89	-	-	-	0.35	0.08	0.07	0.13
24	4-Hydroxy-9-fluorenone	34.51	196.0518	C13H8O2	Fluorene	89	0.03	0.04	0.02	0.24	0.12	0.11	0.07
25	Cyclopenta(def)phenanthrenone	36.23	204.0568	C15H8O	Anthracene	96	-	0.02	-	0.81	0.23	0.09	0.13
26	9,10-Anthracenedione, 2-methyl-	37.31	165.0698	C15H10O2	Anthraquinone	95	0.01	-	-	0.12	0.07	0.04	0.05
27	9-Anthracenecarboxaldehyde	37.51	206.0722	C15H10O	Anthraquinone	90	-	-	-	0.08	0.03	0.01	0.01
28	Benzo[b]naphtho[2,3-d]furan	37.55	218.0724	C16H10O	Anthraquinone	96	-	-	-	0.96	0.15	0.06	0.05
29	9-Anthracenecarboxaldehyde	37.73	206.0722	C15H10O	Anthraquinone	96	-	-	-	0.23	0.10	0.03	0.04
30	Benzo[kl]xanthene	37.85	218.0724	C16H10O	Xanthone	94	-	-	-	0.34	0.08	0.03	0.03
31	Benzo(b)naphtho(1,2-d)furan	38.12	218.0724	C16H10O	Xanthone	93	-	-	-	0.50	0.11	0.04	0.03
32	Benzo[b]naphtho[2,3-d]furan	38.53	218.0724	C16H10O	Anthraquinone	94	-	-	-	0.18	0.06	0.02	-
33	2H-Phenanthro[9,10-b]pyran (isomer)	39.19	232.0879	C17H12O	Anthraquinone	91	-	-	-	0.05	0.06	0.02	0.02
34	2H-Phenanthro[9,10-b]pyran(isomer)	39.75	232.0879	C17H12O	Anthraquinone	88	-	-	-	0.18	0.06	0.02	0.02
35	4-tert-Butyl-benzophenone	40.48	223.1116	C17H18O	Anthraquinone	89	-	-	-	0.04	0.04	0.01	0.01
36	4-Phenyldibenzofuran	40.64	244.0882	C18H12O	Anthraquinone	93	-	-	-	0.07	0.02	0.01	0.01
37	7H-Benz[de]anthracen-7-one (isomer)	41.49	230.0725	C17H10O	Anthraquinone	97	0.01	0.00	0.00	0.32	0.11	0.04	0.03
38	11H-Benzo[a]fluoren-11-one	42.04	230.0725	C17H10O	Anthraquinone	96	-	-	-	0.12	0.04	0.01	0.01
39	7H-Benz[de]anthracen-7-one (isomer)	42.47	230.0725	C17H10O	Anthraquinone	97	-	-	-	0.39	0.12	0.03	0.03
40	Benzo[1,2-b:5,4-b']bisbenzofuran	43.55	258.0676	C18H10O2	Anthraquinone	97	-	-	-	0.08	0.02	0.01	0.01
41	7H-Benz[de]anthracen-7-one	43.78	230.0725	C17H10O	Anthraquinone	97	0.01	0.00	0.00	0.93	0.24	0.05	0.03
42	Naphtho[2,1,8,7-klmn]xanthene	44.04	242.0726	C18H10O	Anthraquinone	94	-	-	-	0.15	0.05	0.02	0.01
43	Dinaphtho[1,2-b:1',2'-d]furan	48.26	268.0883	C20H12O	Anthraquinone	92	-	-	-	0.06	-	-	-
						Sum	0.94	1.1	0.40	51	15	5.3	5.8

PAH, S-containing PAH

1	Thioxanthene	32.46	198.0500	C13H10S	Xanthone	82	0.013	0.009	-	0.04	-	0.06	-
2	1,9-Dihydropyrene	32.62	204.0930	C16H14	Pyrene	92	-	-	-	0.06	-	-	-
3	Phenanthrene, 1-methyl-	33.30	192.0925	C15H12	Phenanthrene	97	-	-	-	0.20	0.03	0.01	0.02
4	1H-Indene, 1-phenyl-	33.41	192.0925	C15H12	Phenanthrene	96	-	-	-	0.33	0.05	0.02	0.02
5	Phenanthrene, 9-methyl-	33.58	192.0925	C15H12	Phenanthrene	89	-	-	-	0.06	0.01	0.00	0.00
6	4H-Cyclopenta[def]phenanthrene	33.71	189.0697	C15H10	Phenanthrene	96	-	-	-	0.49	0.06	0.02	0.03
7	5H-Dibenzo[a,d]cycloheptene	33.78	192.0925	C15H12	Anthracene	90	-	-	-	0.20	0.03	0.01	0.02
8	Anthracene, 2-methyl-	33.88	192.0925	C15H12	Anthracene	93	-	-	-	0.39	0.06	0.02	0.01
9	Naphthalene, 2-phenyl- (isomer)	34.81	204.0930	C16H12	Naphthalene	97	-	-	-	0.65	0.08	0.03	0.02
10	Phenanthrene, 4,5-dimethyl-(isomer)	35.44	191.0853	C16H14	Phenanthrene	91	-	-	-	0.11	0.03	0.01	0.02
11	Phenanthrene, 4,5-dimethyl-(isomer)	35.52	191.0853	C16H14	Phenanthrene	91	-	-	-	0.04	0.01	0.00	-
12	Naphthalene, 2-phenyl- (isomer)	35.90	204.0930	C16H12	Naphthalene	94	-	-	-	0.08	0.02	0.01	0.00
13	Phenanthrene, 2,3-dimethyl-(isomer)	35.98	206.1086	C16H14	Phenanthrene	95	-	-	-	0.11	0.02	0.01	0.02
14	Phenanthrene, 3,6-dimethyl- (isomer)	36.09	206.1086	C16H14	Phenanthrene	93	-	-	-	0.09	0.01	0.00	0.01
15	Phenanthrene, 3,6-dimethyl- (isomer)	36.20	206.1086	C16H14	Phenanthrene	96	0.001	0.001	0.001	0.25	0.05	0.01	0.01
16	9-Ethyl-10-methylanthracene	36.63	205.1010	C17H16	Anthracene	95	-	-	-	0.14	0.08	0.01	0.01
17	Acephenanthrylene	36.88	202.0772	C16H10	Fluoranthene	97	-	-	-	2.16	0.36	0.09	0.07
18	Naphthalene, 2-(phenylmethyl)- (isomer)	36.90	218.1087	C17H14	Naphthalene	95	-	-	-	0.11	0.03	0.01	0.01
19	Naphthalene, 2-(phenylmethyl)- (isomer)	37.05	218.1087	C17H14	Naphthalene	93	-	-	-	0.06	0.02	0.00	0.01
20	Pyrene, 4,5-dihydro-	37.53	204.0930	C16H12	Fluoranthene	94	-	-	-	0.16	0.04	0.01	0.01
21	Naphthalene, 1-(4-methylphenyl)-	37.55	218.1087	C17H14	Fluoranthene	93	-	-	-	0.07	0.04	0.01	-
23	p-Terphenyl	38.10	230.1088	C18H14	Fluoranthene	94	-	-	-	0.09	0.02	0.01	0.00
24	9-Ethyl-10-methylanthracene	38.14	205.1010	C17H16	Anthracene	93	-	-	-	0.25	0.18	0.02	0.04
25	Pyrene, 4-methyl-	38.64	216.0920	C17H12	Pyrene	92	-	-	-	0.28	0.08	0.02	0.02
26	4H-Benz[de]anthracene, 5,6-dihydro-	38.81	218.1087	C17H14	Benz[a]anthracene	94	-	-	-	0.17	0.13	0.03	0.00
27	7H-Benzanthrene	39.02	215.0854	C17H12	Benz[a]anthracene	91	-	-	-	0.74	0.38	0.07	0.07
28	Retene	39.28	219.1167	C18H18	Benz[a]anthracene	97	-	-	-	1.2	1.11	0.08	0.08
29	11H-Benzo[b]fluorene	39.44	215.0854	C17H12	Benz[a]anthracene	94	-	-	-	0.62	0.17	0.03	0.03
30	Pyrene, 1-methyl- (isomer)	39.54	216.0920	C17H12	Pyrene	95	-	-	-	0.27	0.07	0.01	0.02
31	Pyrene, 1-methyl- (isomer)	40.06	216.0920	C17H12	Pyrene	93	-	-	-	0.37	0.10	0.02	0.03
32	Naphthacene	40.16	228.0929	C18H12	Fluoranthene	90	-	-	-	0.08	0.03	0.01	0.00

33	7H-Benzo[c]fluorene	40.22	215.0854	$C_{17}H_{12}$	Fluoranthene	96	-	-	-	0.06	0.09	0.02	0.03
34	1,4-Dimethyl-6-phenyl-naphthalene	40.49	232.1245	$C_{18}H_{16}$	Fluoranthene	93	-	-	-	0.15	0.16	0.02	0.01
35	Benzo[b]naphtho[1,2-d]thiophene	41.93	234.0498	$C_{16}H_{10}S$	Fluoranthene	90	-	-	-	0.03	0.00	0.09	0.00
36	Cyclopenta[cd]pyrene	42.10	226.0773	$C_{18}H_{10}$	Fluoranthene	96	-	-	-	2.6	0.56	0.13	0.36
37	Cyclopenta(cd)pyrene, 3,4-dihydro-	42.14	228.0929	$C_{18}H_{12}$	Fluoranthene	94	-	-	-	0.48	0.14	0.03	0.03
38	1,2-Dihydrobenzo[b]fluoranthene	43.66	253.1008	$C_{20}H_{14}$	Benzo[k]fluoranthene	98	-	-	-	0.06	0.02	0.00	0.01
39	9H-Tribenzo[a,c,e)cycloheptene	45.05	242.1087	$C_{19}H_{14}$	Benzo[k]fluoranthene	97	-	-	-	0.20	0.09	0.01	0.01
40	Benz[a]anthracene, 7-methyl-	45.21	242.1087	$C_{19}H_{14}$	Benz[a]anthracene	97	-	-	-	0.11	0.03	0.01	-
41	Benzo(a)pyrene, 7,8-dihydro-	45.60	239.0853	$C_{20}H_{14}$	Benzo[k]fluoranthene	89	-	-	-	0.24	0.07	0.01	0.01
42	Binaphthalene (isomer)	45.89	254.1088	$C_{20}H_{14}$	Benzo[k]fluoranthene	97	-	-	-	0.09	0.04	0.01	0.00
43	Binaphthalene (isomer)	46.14	254.1088	$C_{20}H_{14}$	Benzo[k]fluoranthene	97	-	-	-	0.14	0.06	0.01	0.02
44	Benzo[j]fluoranthene	48.25	252.0930	$C_{20}H_{12}$	Benzo[k]fluoranthene	98	-	-	-	0.58	0.10	-	-
45	Benzo[e]pyrene	48.88	252.0930	$C_{20}H_{12}$	Benzo[k]fluoranthene	97	0.008	-	-	0.91	0.19	0.03	0.04
						**Sum**	**0.02**	**0.01**	**0.00**	**15**	**4.8**	**0.99**	**1.1**

Table S4. Meteorological data (temperature, RH, wind speed, wind direction) corresponding to the winter (W1–W4) and summer (S1–S3) sampling

Sample ID	W1	W2	W3	W4	S1	S2	S3
Date	2024-11-14	2024-12-24	2025-01-16	2025-01-21	2025-08-11	2025-08-19	2025-08-23
Time	9:30-11:30	9:14-11:14	9:05-11:05	9:30-11:30	10:00-12:00	9:30-11:30	9:30-11:30
t, °C	-8	0	-4	-7	+17	+12	+14
Humidity, %	87	96	92	80	77	73	73
Wind speed (m/s)	1.4	2.8	3.6	3.0	3.1	2.7	2.4
Wind diraction	NW	SSW	SSW	NW	ESE	SSE	SSE

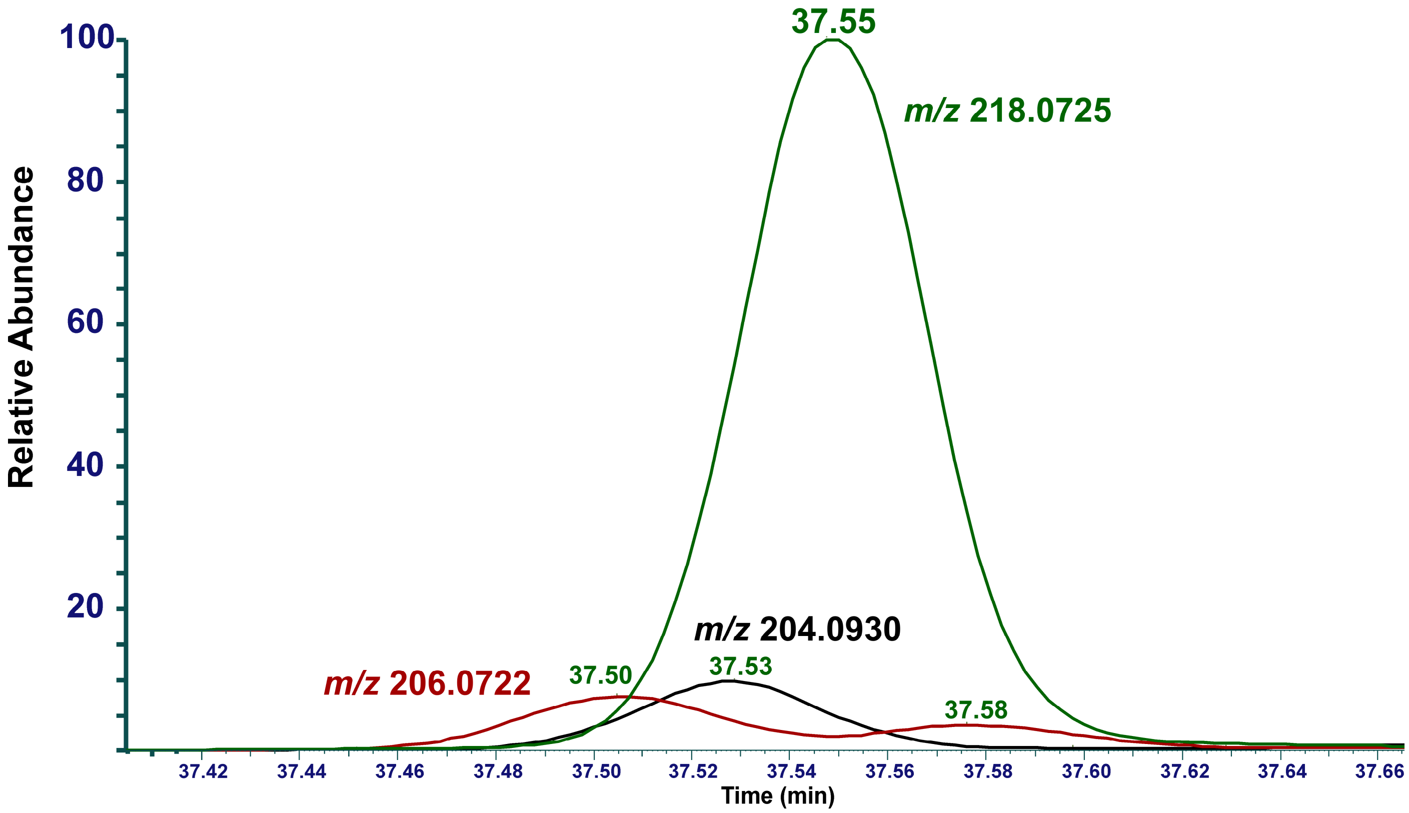

Figure S1 - Zoomed view of the extracted ion chromatograms (XIC) for the 37.4–37.7 min region of sample W1. The chromatograms were extracted with a mass tolerance of ±5 ppm. The figure illustrates the separation of co-eluting PAHs based on their characteristic exact masses (m/z 206.0722 ($C_{15}H_{10}O$), 204.0930 ($C_{16}H_{12}$), 218.0725 ($C_{17}H_{14}$)).

 https://doi.org/10.3390/xxxxx

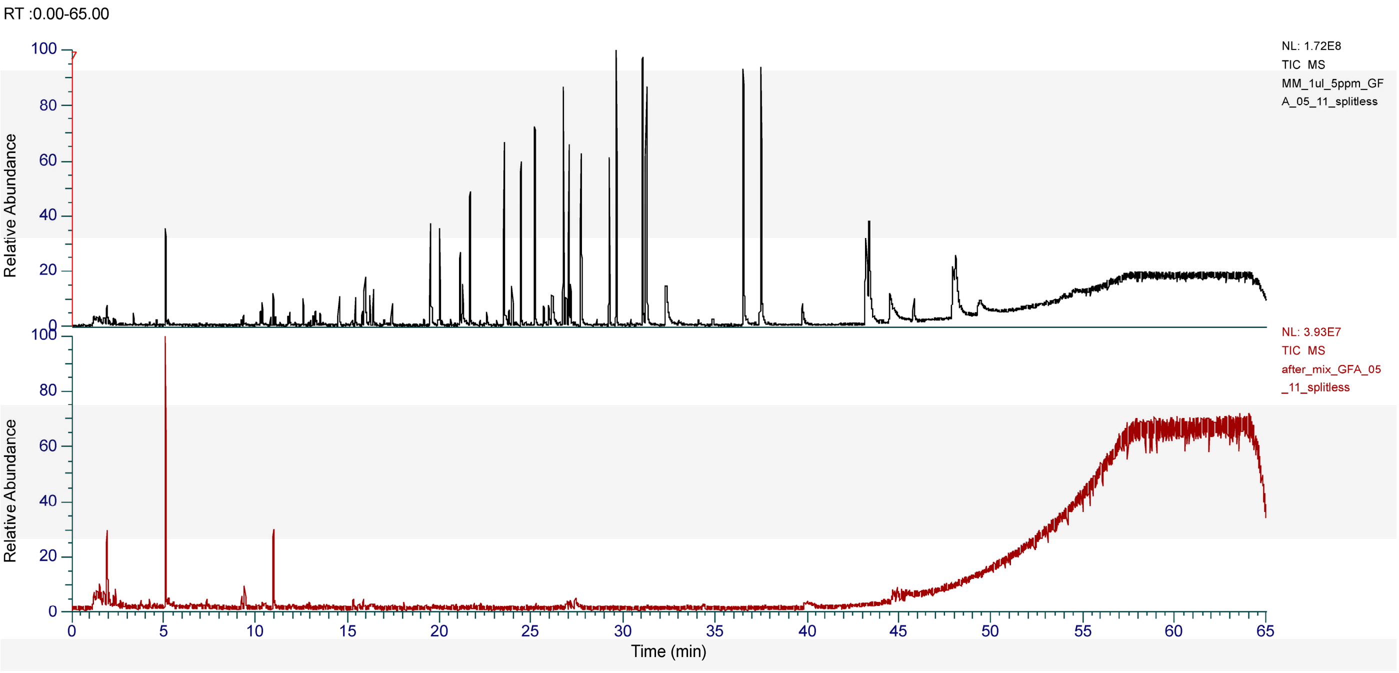

Figure S2 - TIC chromatograms. A standard mixture of target analytes with a concentration of 1 mg/L (black). An empty filter is analyzed immediately after the standard (red). No significant peaks were detected in the blank (>0.1% of the standard signal)

Table S5 – Relative standard deviations (RSD) for 72 target compounds after three repetitions of thermal desorption from the filter (5 ng of each compound)

№	tr, min	Compounds	SD	RSD, %
**N-compounds**				
1	3.35	N-Nitrosodimethylamine	**102418.2**	**6.5**
2	3.39	Pyridine	**850035.5**	**9.9**
3	10.72	Aniline	**212928.3**	**6.6**
4	13.5	N-Nitroso-di-n-propylamine	**119888.2**	**3.2**
5	15.12	2-Nitrophenol	**38730.02**	**4.8**
6	16.95	4-Chloroaniline	**795055.2**	**10.6**
7	21.58	2-Nitroaniline	**611924.5**	**6.3**
8	22.17	1,4-Dinitrobenzene	**111397.1**	**10.0**
9	22.42	1,3-Dinitrobenzene	**153017.2**	**7.4**
10	22.62	2,6-Dinitrotoluene	**1204215**	**7.3**
11	22.74	1,2-Dinitrobenzene	**80284.52**	**10.6**
12	23.2	3-Nitroaniline	**355082.9**	**9.1**
13	24.13	2,4-Dinitrotoluene	**1080798**	**10.1**
14	25.55	4-Nitroaniline	**128397.8**	**6.7**
15	25.97	Diphenylamine	**3926692**	**4.6**
16	26.07	Azobenzene	**1463272**	**7.9**
17	29.88	Carbazole	**1471150**	**1.7**

		**Phenols**		
18	10.9	Phenol	**1062923**	**8.1**
19	12.55	Benzyl alcohol	**463373.3**	**7.0**
20	13.05	2-Methylphenol	**289734.9**	**6.8**
21	13.67	3+4-Methylphenol	**61587.59**	**2.1**
22	13.67			
23	14.87	Isophorone	**1935179**	**8.2**
24	15.6	2,4-Dimethylphenol	**1153632**	**10.3**
		**Halogenated SVOCs**		
25	11.07	Bis(2-chloroethyl)ether	**254998.3**	**5.4**
26	11.08	2-Chlorophenol	**1035843**	**5.5**
27	13.57	Hexachloroethane	**92822.58**	**9.6**
28	15.92	Bis(2-chloroethoxy)methane	**593459.8**	**9.9**
29	16.07	2,4-Dichlorophenol	**2000636**	**6.8**
30	19.03	4-Chloro-3-methylphenol	**1023872**	**7.9**
31	20.52	2,4,6-Trichlorophenol	**2352011**	**10.1**
32	20.62	2,4,5-Trichlorophenol	**1511153**	**8.8**
33	21.02	2-Chloronaphthalene	**1335472**	**1.5**
34	24.39	2,3,5,6-Tetrachlorophenol	**576182.4**	**6.9**
35	24.55	2,3,4,6-Tetrachlorophenol	**951947.5**	**7.8**
36	25.49	4-Chlorophenyl phenyl ether	**1967960**	**2.4**
37	27.33	4-Bromophenyl phenyl ether	**1511514**	**2.5**
38	27.37	Hexachlorobenzene	**3221143**	**3.9**
39	28.23	Pentachlorophenol	**192285.3**	**8.7**
		**PAHs**		
40	16.51	Naphthalene	**1296333**	**3.7**
41	19.21	2-Methylnaphthalene	**624907.1**	**1.2**
42	19.56	1-Methylnaphthalene	**1422683**	**2.6**
43	22.55	Acenaphthylene	**2121366**	**1.3**
44	23.26	Acenaphthene	**2209244**	**2.5**
45	23.94	Dibenzofuran	**1170617**	**0.7**
46	25.25	Fluorene	**3645461**	**2.7**
47	28.89	Phenanthrene	**2240377**	**1.0**
48	29.09	Anthracene	**5190146**	**2.5**
49	33.47	Fluoranthene	**1306298**	**0.5**
50	34.27	Pyrene	**552649.6**	**0.2**
51	38.97	Benz[a]anthracene	**262980.8**	**0.2**
52	39.09	Chrysene	**1969377**	**1.0**
53	42.94	Benzo[b]fluoranthene	**2627605**	**3.3**
54	43.03	Benzo[k]fluoranthene	**3792427**	**2.3**
55	43.93	Benzo[a]pyrene	**5756059**	**8.1**
56	53.95	Imdeno(1,2,3-cd)pyrene	**1907552**	**9.9**
57		Dibenz(a,h)anthracene	**1300183**	**8.6**

58	48.27	Benzo[g,h,i]perylene	**3709464**	**10.4**
		**Phthalates**		
59	22.51	Dimethylphthalate	**1427235**	**8.6**
60	25.32	Diethylphthalate	**2212222**	**5.1**
61	31.78	Di-*n*-butyl phthalate	**48702.36**	**2.1**
62	37.49	Benzyl butyl phthalate	**852319.8**	**10.0**
63	38.17	Bis(2-ethylhexyl)adipate	**6748.461**	**8.1**
64	42.42	Di-*n*-octyl phthalate	**240236.1**	**10.4**
		**Oxy-PAHs**		
65	22.63	1.4-Naphthoquinone	**1449333**	**1.6**
66	25.12	1-Naphthol	**1279364**	**8.0**
67	28.21	2-Nitronaphthalene	**4886866**	**2.8**
68	32.76	Xanthone	**1911895**	**8.9**
69	32.99	Acenaphthenequinone	**1212105**	**8.8**
70	34.54	Anthrone	**63378.26**	**8.2**
71	34.94	Anthraquinone	**2397672**	**1.1**
72	36.26	1.8-Naphthalic anhydride	**689989.8**	**8.3**

https://doi.org/10.3390/xxxxx
